# Supplementary material for: Circulating trans fatty acids are associated with prostate cancer in Ghanaian and American men
Source: Nat Commun. 2023 Jul 19;14:4322. doi: 10.1038/s41467-023-39865-9 (PMC10356769; doi:10.1038/s41467-023-39865-9)
Supplement: Supplementary file 1 — Supplementary information [file 41467_2023_39865_MOESM1_ESM.pdf]

# Supplementary Figures

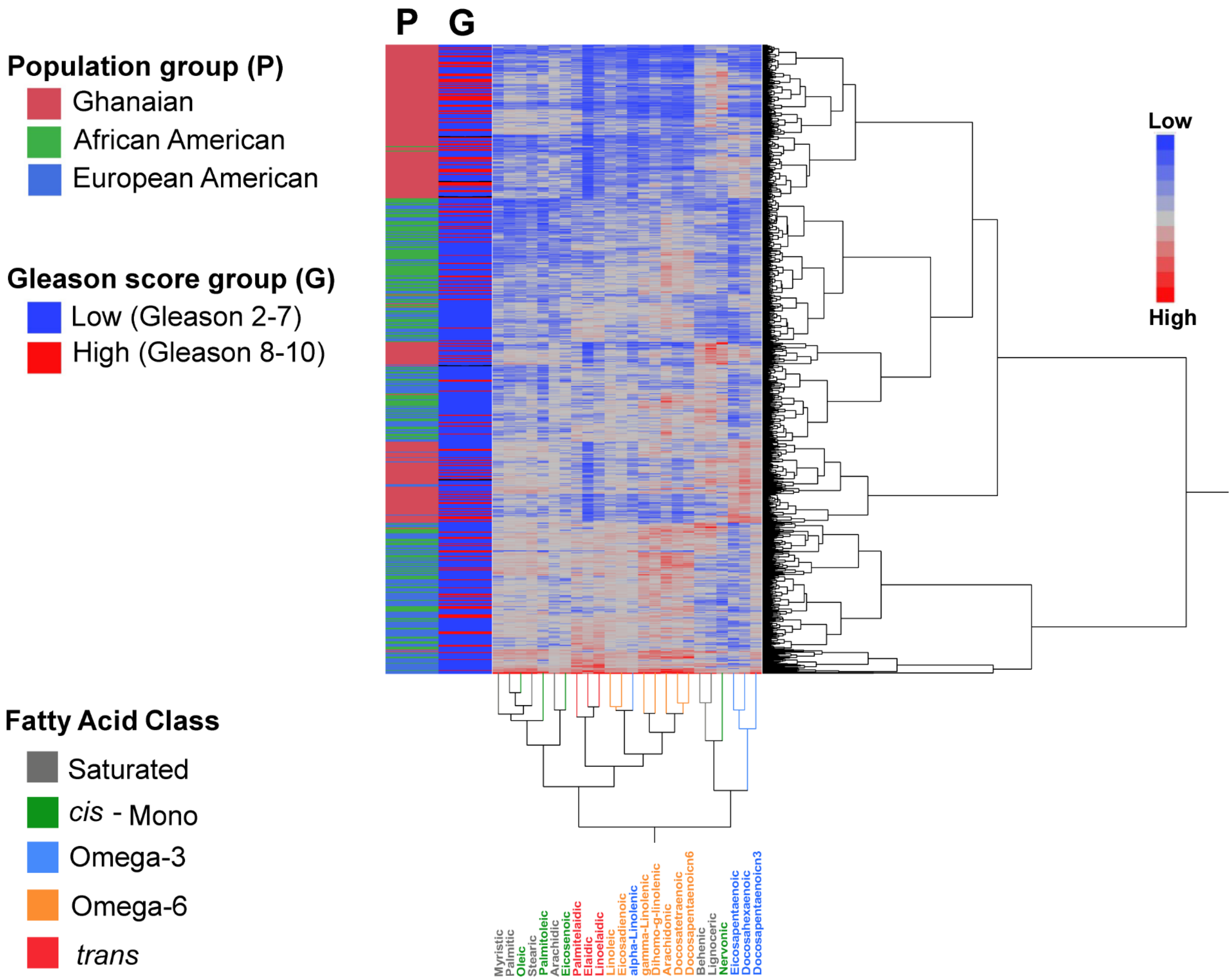


**Supplementary Figure 1. Circulating fatty acid levels by fatty acid class, population group, and Gleason score among cases in the NCI-Maryland and NCI-Ghana cohorts.** Heatmaps depicting unsupervised hierarchical clustering using absolute (mean, µg/mL) concentrations from individual fatty acids in prostate cancer cases (n=1,431). Green indicates lower concentrations and red indicates higher concentrations for each participant. Samples were labeled by population group, P, [Ghanaian (red, n=1,243), African American (green, n=788), and European American (blue, n=903) men] and dichotomized Gleason score, G, [≤ 7 (blue, n=1,081); ≥ 8 (red, n=343)]. Individual fatty acids were color-coded by fatty acid class (Saturated, grey; *cis*-Monounsaturated, green; Omega-3, blue; Omega-6, orange; and *trans*, red). Source data are provided as a Source Data file.

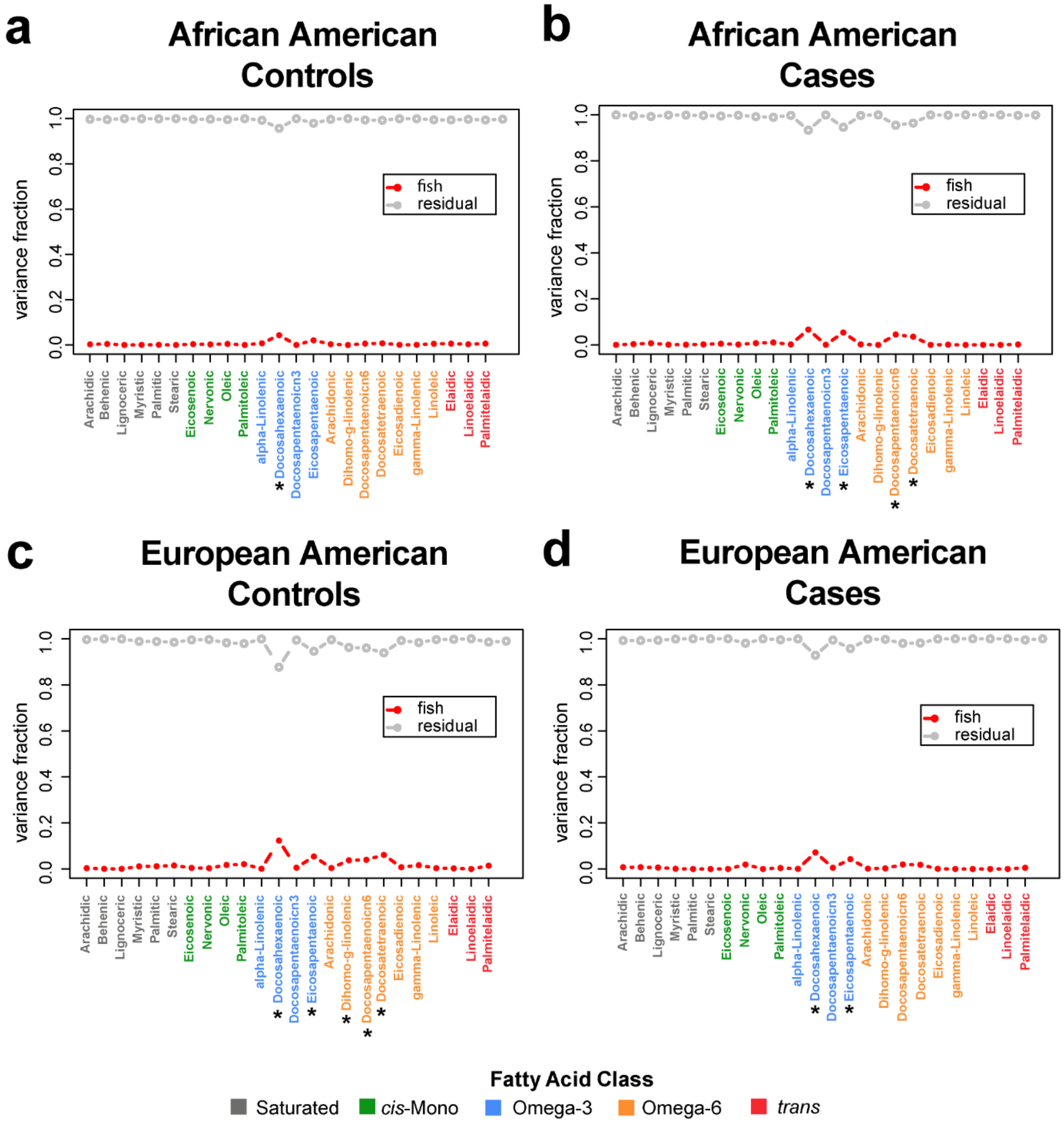


**Supplementary Figure 2. Influence of fish consumption on circulating levels of fatty acids among African American and European American men.** Variance analysis for the levels of each of the 24 fatty acids are assessed as a function of fish consumption frequency among **(a)** African American controls (n=380), **(b)** African American cases (n=379), **(c)** European American controls (n=460), and **(d)** European American cases (n=428). Red dotted line represents the proportion of variance in the levels of the fatty acids that can be explained by fish consumption while the grey line represents the residual variance that remains to be explained by other factors. Asterisks (*) indicate fatty acids that are significantly explained by how often the men ate fish using an Analysis of Variance statistical test with a Bonferroni-adjusted significance threshold of $P = 0.002$ to account for multiple testing. Fatty acids are color coded to indicate their fatty acid class (Saturated, grey; *cis*-Monounsaturated, green; Omega-3, blue; Omega-6, orange; and *trans*, red). Source data are provided as a Source Data file.

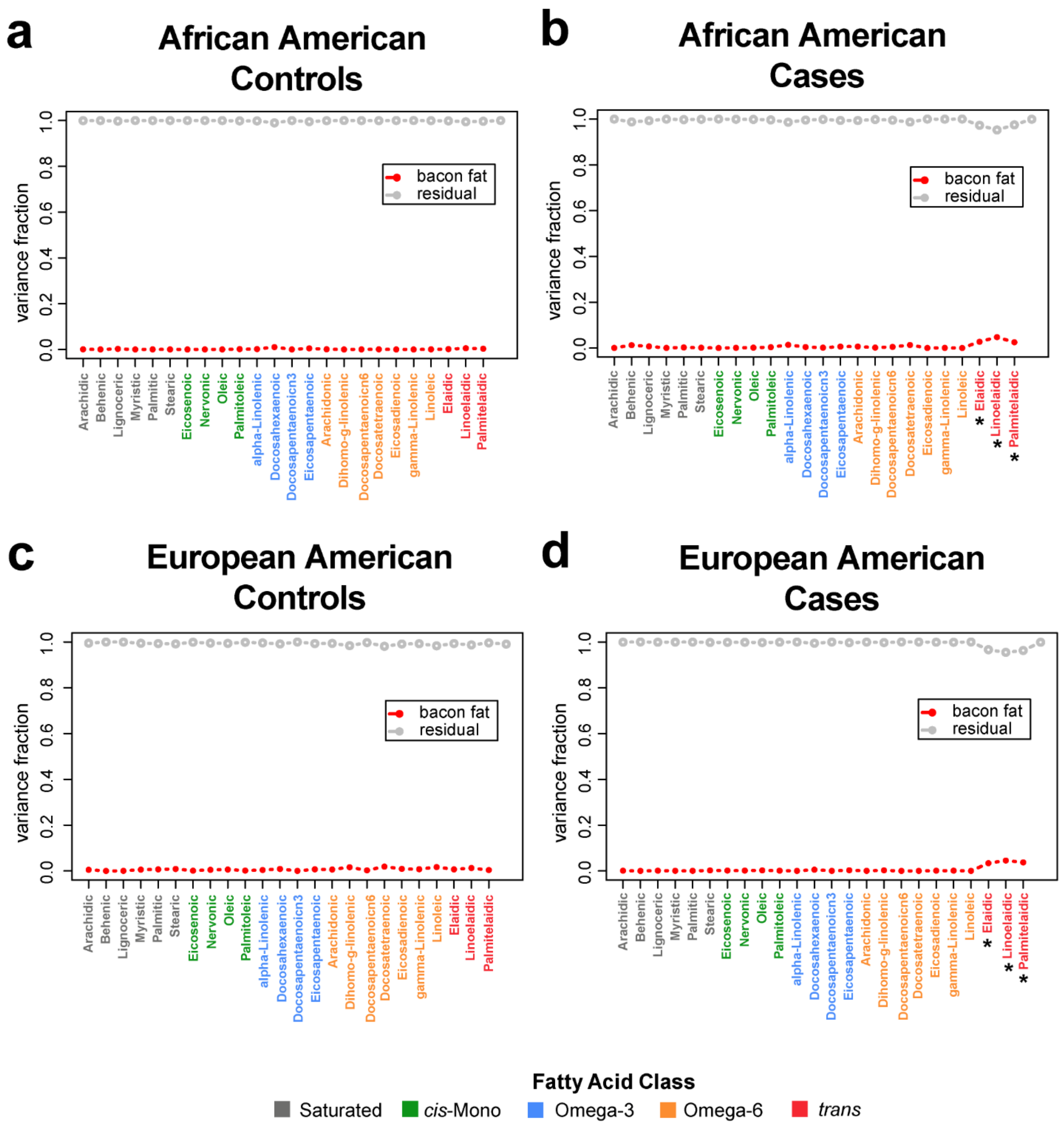


**Supplementary Figure 3. Influence of bacon fat on circulating levels of fatty acids among African American and European American men.** Variance analysis for the levels of each of the 24 fatty acids are assessed as a function of how often **(a)** African American controls (n=380), **(b)** African American cases (n=404), **(c)** European American controls (n=463), and **(d)** European American cases (n=436) had bacon-fat or drippings in their meals. Red dotted line represents the proportion of variance in the levels of the fatty acids that can be explained by having bacon-fat or drippings in their meals while the grey line represents the residual variance that remains to be explained by other factors. Asterisks (*) indicate fatty acids that are significantly explained by bacon-fat consumption frequency using an Analysis of Variance statistical test and a Bonferroni-adjusted significance threshold of $P = 0.002$ to account for multiple testing. Fatty acids are color coded to indicate their fatty acid class (Saturated, grey; *cis*-Monounsaturated, green; Omega-3, blue; Omega-6, orange; and *trans*, red). Source data are provided as a Source Data file.

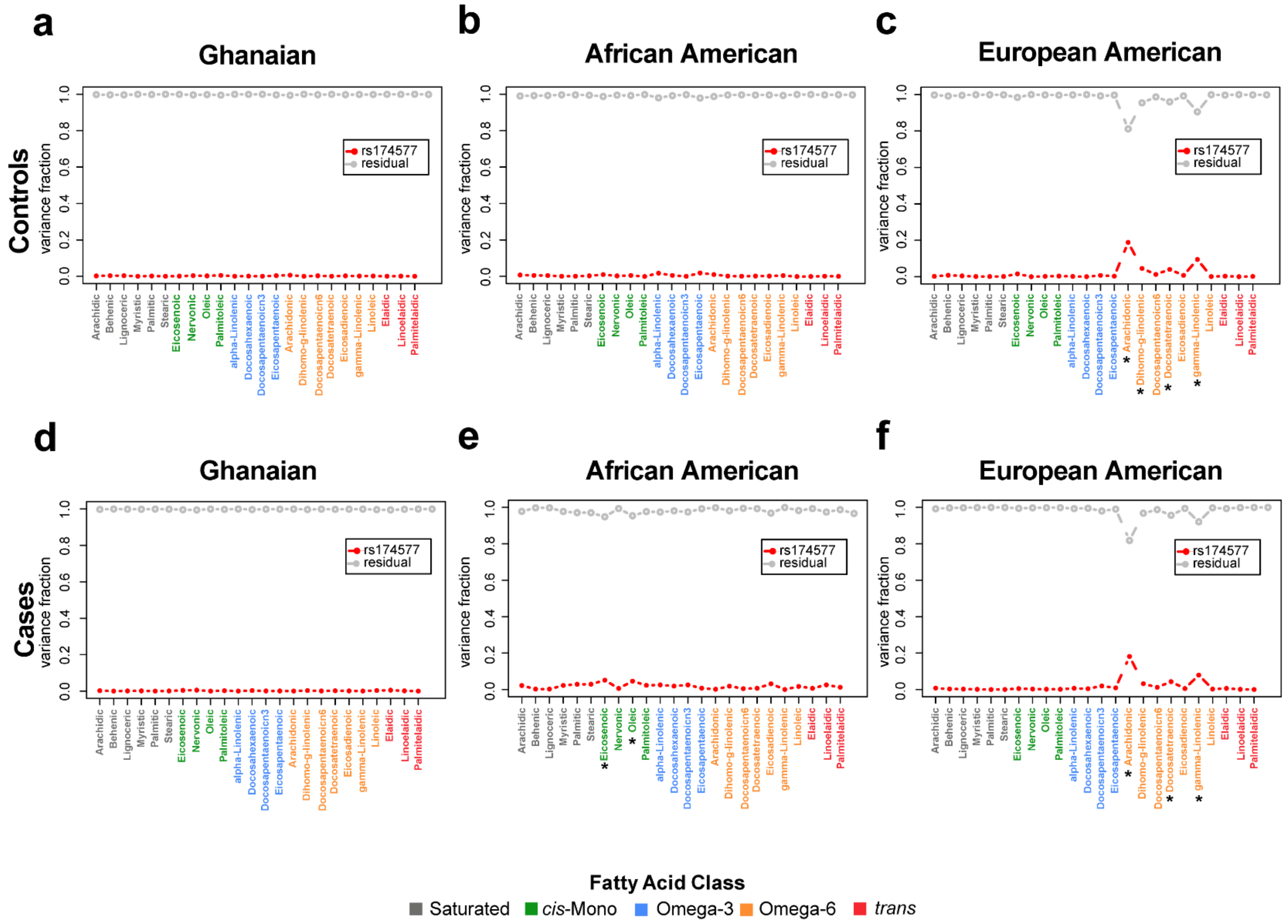


**Supplementary Figure 4. Influence of *FADS2* SNP, rs174577, on circulating levels of fatty acids.** Variance analysis for the levels of each of the 24 fatty acids are assessed as a function of rs174577 among **(a)** Ghanaian controls (n=600), **(b)** African American controls (n=349), **(c)** European American controls (n=392), **(d)** Ghanaian cases (n=511), **(e)** African American cases (n=344), and **(f)** European American cases (n=361). Red dotted line represents the proportion of variance in the levels of the fatty acids that can be explained by rs174577 while the grey line represents the residual variance that remains to be explained by other factors. Asterisks (*) indicate variances in fatty acid levels that are significantly explained by the SNP using an Analysis of Variance statistical test. Bonferroni-adjusted significance threshold of $P = 0.002$ is used to account for multiple testing. Fatty acids are color coded to indicate their fatty acid class (Saturated, grey; *cis*-Monounsaturated, green; Omega-3, blue; Omega-6, orange; and *trans*, red). SNP = Single Nucleotide Polymorphism. Source data are provided as a Source Data file.

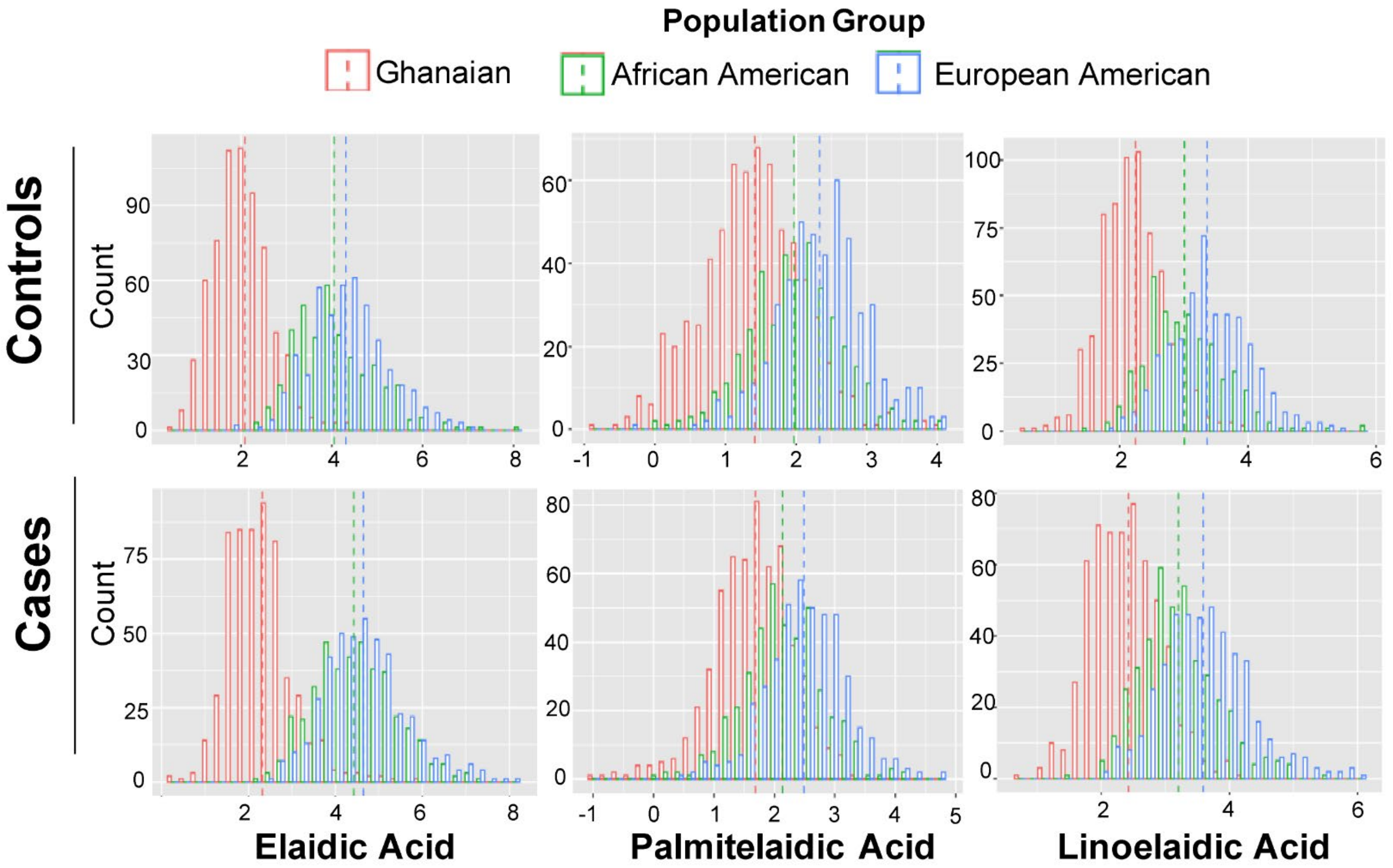


**Supplementary Figure 5**. **Frequency distribution of *trans* fatty acids by population group**. Frequency distribution of $\log_2$ transformed absolute (mean, μg/mL) concentrations of three *trans* fatty acids (elaidic, palmitelaidic, and linoelaidic) in controls (top, n=1,503) and cases (bottom, n=1,431) by population group. Ghanaian (red), African American (green), European American (blue) men. Source data are provided as a Source Data file.

## Supplementary Tables 1-17

**Supplementary Table 1.** Characteristics of cases and controls in the NCI-Ghana and NCI-Maryland Studies

| Demographics | Controls | | | Cases | | |
|---|---|---|---|---|---|---|
| | Ghanaian (n=658) | African American (n=381) | European American (n=464) | Ghanaian (n=585) | African American (n=407) | European American (n=439) |
| Age[a] | | | | | | |
| Median (IQR[b]) in years | 59 (11) | 64 (10) | 66.5 (13) | 70 (11) | 63 (11) | 65 (11) |
| BMI | | | | | | |
| Mean(SD[c]) in kg/m2 | 24.3 (4.4) | 29.7 (5.5) | 27.8 (4.9) | 25.6 (4.7) | 28.0 (5.2) | 28.0 (4.3) |
| Education for NCI-MD and NCI-Ghana, *N* (%) | | | | | | |
| No formal education | 76 (12) | NA | NA | 43 (7) | NA | NA |
| Elementary/primary | 47 (7) | 2 (1) | 2 (<1) | 17 (3) | 4 (1) | 1 (<1) |
| Middle/junior secondary | 293 (45) | 14 (4) | 7 (2) | 162 (28) | 24 (6) | 17 (4) |
| High School/GED/senior secondary | 126 (19) | 94 (25) | 76 (16) | 117 (20) | 163 (40) | 95 (22) |
| Higher | 86 (13) | 270 (71) | 379 (82) | 228 (39) | 215 (53) | 326 (74) |
| Did not provide | 30 (5) | 1 (<1) | - | 18 (3) | 1 (<1) | - |
| Annual household income, *N* (%) | | | | | | |
| Less than $10,000 | NA | 18 (5) | 5 (1) | NA | 62 (15) | 17 (4) |
| $10,000- $29,999 | NA | 65 (17) | 39 (8) | NA | 138 (34) | 83 (19) |
| $30,000 - $59,999 | NA | 79 (21) | 111 (24) | NA | 96 (24) | 92 (21) |
| $60,000-$90,000 | NA | 91 (24) | 106 (23) | NA | 41 (10) | 88 (20) |
| Greater than $90,000 | NA | 89 (23) | 175 (38) | NA | 32 (8) | 133 (30) |
| Don't know/Refused | NA | 30 (8) | 17 (4) | NA | 16 (4) | 14 (3) |
| Did not provide | NA | 9 (2) | 11 (2) | NA | 22 (5) | 12 (3) |
| **Baseline Health Factors** | | | | | | |
| Smoking status[d], *N* (%) | | | | | | |
| Never | 347 (53) | 152 (40) | 194 (42) | 386 (66) | 116 (29) | 176 (40) |
| Former | 188 (29) | 157 (41) | 221 (48) | 180 (31) | 155 (38) | 195 (44) |
| Current | 95 (14) | 69 (18) | 43 (9) | 10 (2) | 133 (33) | 66 (15) |
| Total | 28 (4) | 3 (1) | 6 (1) | 9 (2) | 3 (1) | 2 (<1) |
| Diabetes, *N* (%) | | | | | | |
| No | 606 (92) | 265 (70) | 385 (83) | 472 (81) | 293 (72) | 373 (85) |
| Yes | 45 (7) | 116 (30) | 79 (17) | 107 (18) | 114 (28) | 66 (15) |
| Did not provide | 7 (1) | - | - | 6 (1) | - | - |
| Regular aspirin use, *N* (%) | | | | | | |
| No | 574 (87) | 183 (48) | 175 (38) | 423 (72) | 237 (58) | 195 (44) |
| Yes | 66 (10) | 198 (52) | 289 (62) | 91 (16) | 170 (42) | 244 (56) |
| Did not provide | 18 (3) | - | - | 71 (12) | - | - |
| Gleason score, *N* (%) | | | | | | |
| Gleason score 2-6 (low grade) | NA | NA | NA | 179 (31) | 164 (40) | 195 (44) |
| Gleason score 7 (intermediate grade) | NA | NA | NA | 202 (35) | 173 (43) | 168 (38) |
| Gleason score 8-10 (high grade) | NA | NA | NA | 197 (34) | 70 (17) | 76 (17) |
| Unavailable | NA | NA | NA | 7 (1) | - | - |
| Stage[e], *N* (%) | | | | | | |
| I | NA | NA | NA | NA | 64 (16) | 100 (23) |
| II | NA | NA | NA | NA | 289 (71) | 271 (62) |
| III | NA | NA | NA | NA | 24 (6) | 44 (10) |
| IV | NA | NA | NA | NA | 30 (7) | 24 (5) |
| NCCN risk groups[f], *N* (%) | | | | | | |
| Low | NA | NA | NA | NA | 63 (15) | 101 (23) |
| Intermediate | NA | NA | NA | NA | 223 (55) | 215 (49) |
| High/Very high | NA | NA | NA | NA | 95 (23) | 102 (23) |
| Regional/ Metastatic | NA | NA | NA | NA | 26 (6) | 21 (5) |
| PSA[g] | | | | | | |
| Median (IQR) in ng/ml | 1 (1.5) | 0.4 (0.6) | 0.4 (0.6) | 52 (101.9) | 6.9 (7.6) | 5.9 (4.8) |

[a] Age at recruitment
[b] IQR: Interquartile range
[c] SD: Standard deviation
[d] Smoking status describes cigarette smoking
[e] Pathologically confirmed using American Joint Committee on Cancer (AJCC) 7th Edition
[f] Risk stratification based on the 2019 National Comprehensive Cancer Network (NCCN) guideline (see methods)
[g] PSA: Prostate specific antigen

**Supplementary Table 2.** Individual fatty acids (n = 24) grouped into five distinct classes

| Fatty acid | Nomenclature[a] | Fatty acid class |
|---|---|---|
| Arachidic | 20:0 | Saturated |
| Behenic | 22:0 | Saturated |
| Lignoceric | 24:0 | Saturated |
| Myristic | 14:0 | Saturated |
| Palmitic | 16:0 | Saturated |
| Stearic | 18:0 | Saturated |
| Eicosenoic | 20:1n-9 | *cis* -Monounsaturated |
| Nervonic | 24:1n-9 | *cis* -Monounsaturated |
| Oleic | 18:1n-9 | *cis* -Monounsaturated |
| Palmitoleic | 16:1n-7 | *cis* -Monounsaturated |
| alpha-Linolenic (ALA) | 18:3n-3ccc | Omega-3 |
| Docosahexaenoic (DHA) | 22:6n-3 | Omega-3 |
| Docosapentaenoic - n3 (DPA) | 22:5n-3 | Omega-3 |
| Eicosapentaenoic (EPA) | 20:5n-3 | Omega-3 |
| Arachidonic (AA) | 20:4n-6 | Omega-6 |
| Dihomo-g-linolenic (DGLA) | 20:3n-6 | Omega-6 |
| Docosapentaenoic - n6 | 22:5n-9 | Omega-6 |
| Docosatetraenoic | 22:4n-9 | Omega-6 |
| Eicosadienoic | 20:2n-9 | Omega-6 |
| gamma-Linolenic (GLA) | 18:3n-6 | Omega-6 |
| Linoleic (LA) | 18:2n-6 | Omega-6 |
| Elaidic | 18:1n-9t | *trans* -Monounsaturated |
| Palmitelaidic | 16:1n-7t | *trans* -Monounsaturated |
| Linoelaidic | 18:2n-6t, 9t | *trans* -Polyunsaturated |

[a]Number of carbon atoms: number of double bonds followed by the position of the double bond on the fatty acid carbon chain. The letter t is added if the double bond is in *trans* configuration.

**Supplementary Table 3.** Median absolute concentrations for 24 fatty acids in Ghanaian, African American, and European American men without prostate cancer

| | | Ghanaian (n=658) | African American (n=381) | European American (n=464) | |
|---|---|---|---|---|---|
| **Fatty acid** | **Fatty acid class** | **Median (IQR)** | **Median (IQR)** | **Median (IQR)** | ***P* value*** |
| Arachidic | Saturated | 3.0 (1.1) | 2.7 (1.3) | 3.0 (1.4) | 0.0006 |
| Behenic | Saturated | 4.6 (2.3) | 5.2 (2.6) | 5.3 (2.2) | $3.0 \times 10^{-11}$ |
| Lignoceric | Saturated | 5.2 (2.4) | 4.7 (2.0) | 4.8 (1.9) | $7.4 \times 10^{-7}$ |
| Myristic | Saturated | 23.8 (18.4) | 19.0 (17.2) | 28.2 (23.5) | $6.0 \times 10^{-16}$ |
| Palmitic | Saturated | 681.9 (206.2) | 621.1 (244.7) | 698.4 (280.8) | $1.2 \times 10^{-8}$ |
| Stearic | Saturated | 194.5 (57.3) | 222.4 (74.2) | 231.1 (70.3) | $1.0 \times 10^{-33}$ |
| Eicosenoic | *cis* -Monounsaturated | 4.4 (1.8) | 4.6 (2.2) | 5.2 (2.4) | $9.0 \times 10^{-14}$ |
| Nervonic | *cis* -Monounsaturated | 6.9 (3.7) | 5.6 (2.6) | 5.4 (2.5) | $8.0 \times 10^{-30}$ |
| Oleic | *cis* -Monounsaturated | 608.2 (208.2) | 573.0 (250.0) | 662.2 (307.0) | $2.0 \times 10^{-10}$ |
| Palmitoleic | *cis* -Monounsaturated | 55.3 (42.0) | 31.5 (29.9) | 46.5 (36.6) | $7.0 \times 10^{-35}$ |
| alpha-Linolenic | Omega-3 | 7.5 (4.2) | 17.2 (11.3) | 19.9 (14.7) | $3.0 \times 10^{-173}$ |
| Docosahexaenoic | Omega-3 | 143.4 (58.0) | 55.2 (27.5) | 54.2 (30.8) | $8.0 \times 10^{-210}$ |
| Docosapentaenoic - n3 | Omega-3 | 20.5 (9.9) | 14.1 (7.4) | 16.5 (6.5) | $1.0 \times 10^{-44}$ |
| Eicosapentaenoic | Omega-3 | 60.5 (45.9) | 16.5 (14.0) | 21.0 (19.4) | $5.0 \times 10^{-158}$ |
| Arachidonic | Omega-6 | 161.5 (56.3) | 268.4 (92.0) | 248.5 (80.1) | $5.0 \times 10^{-146}$ |
| Dihomo-g-linolenic | Omega-6 | 33.1 (15.7) | 37.9 (16.1) | 43.6 (18.8) | $3.0 \times 10^{-39}$ |
| Docosapentaenoic - n6 | Omega-6 | 3.8 (2.3) | 5.4 (3.5) | 4.8 (2.9) | $8.0 \times 10^{-32}$ |
| Docosatetraenoic | Omega-6 | 3.5 (1.9) | 7.7 (3.7) | 7.1 (3.8) | $8.0 \times 10^{-159}$ |
| Eicosadienoic | Omega-6 | 5.7 (1.9) | 7.3 (2.7) | 7.7 (3.1) | $1.0 \times 10^{-66}$ |
| gamma-Linolenic | Omega-6 | 9.5 (5.9) | 14.7 (9.1) | 16.1 (9.8) | $9.0 \times 10^{-75}$ |
| Linoleic | Omega-6 | 693.6 (226.4) | 900.7 (292.4) | 947.4 (320.0) | $2.0 \times 10^{-85}$ |
| Elaidic | *trans* -Monounsaturated | 4.1 (2.4) | 15.3 (14.2) | 19.5 (15.3) | $2.0 \times 10^{-223}$ |
| Palmitelaidic | *trans* -Monounsaturated | 2.7 (1.7) | 4.0 (2.4) | 5.0 (2.8) | $1.0 \times 10^{-89}$ |
| Linoelaidic | *trans* -Polyunsaturated | 4.7 (2.2) | 7.7 (4.5) | 9.9 (6.1) | $3.0 \times 10^{-153}$ |

*Kruskal-Wallis rank sum test for significance testing; Bonferroni adjusted threshold for significance is $P = 0.0021$
IQR = Interquartile Range

**Supplementary Table 4.** Median absolute concentrations for fatty acids, when grouped into classes, in Ghanaian, African American, and European American men without prostate cancer

| **Fatty Acid Class** | **Ghanaian (n=658) Median (IQR)** | **African American (n=381) Median (IQR)** | **European American (n=464) Median (IQR)** | ***P* value*** |
|---|---|---|---|---|
| Saturated | 916.3 (264.7) | 883.6 (320.6) | 971.6 (371.5) | $1.9 \times 10^{-6}$ |
| *cis-* Mono | 682.1 (252.8) | 621.4 (266.2) | 723.9 (342.5) | $1.0 \times 10^{-10}$ |
| Omega-*3* | 235.1 (107.0) | 105.2 (52.6) | 114.6 (64.6) | $2.0 \times 10^{-165}$ |
| Omega-6 | 917.9 (268.2) | 1245.8 (352.5) | 1275.5 (363.5) | $4.0 \times 10^{-118}$ |
| Omega 6:3 | 3.9 (1.9) | 11.9 (4.4) | 11.6 (5.3) | $1.0 \times 10^{-218}$ |
| *trans* | 11.7 (5.3) | 27.3 (19.4) | 35.1 (22.5) | $8.0 \times 10^{-208}$ |

*Kruskal-Wallis rank sum test for significance testing; Bonferroni adjusted threshold for significance is $P = 0.0021$

IQR = Interquartile Range

**Supplementary Table 5.** Pairwise comparison of the mean concentrations for each fatty acid class in the three population groups with stratification by case-control status

| Fatty Acid Class | Population Group 1 | Population Group 2 | Control | | Case | |
|---|---|---|---|---|---|---|
| | | | *t* Statistic (95% CI) | *P* value* | *t* Statistic (95% CI) | *P* value |
| Saturated | Ghanaian | European American | 3.31 (27.88, 108.88) | 0.0009 | 4.38 (56.00, 146.86) | $1.3 \times 10^{-5}$ |
| | Ghanaian | African American | -0.69 (-58.09, 27.94) | 0.4919 | -2.45 (-104.38, -11.51) | 0.0145 |
| | African American | European American | 3.54 (37.26, 129.65) | 0.0004 | 6.32 (109.87, 208.88) | $4.0 \times 10^{-10}$ |
| *cis-* Mono | Ghanaian | European American | 3.51 (29.83, 105.42) | 0.0005 | 5.30 (70.76, 153.95) | $1.3 \times 10^{-7}$ |
| | Ghanaian | African American | -1.21 (-64.82, 15.45) | 0.2278 | -2.93 (-105.99, -20.95) | 0.0035 |
| | African American | European American | 4.20 (49.20, 135.41) | $2.8 \times 10^{-5}$ | 7.61 (130.50, 221.16) | $5.0 \times 10^{-14}$ |
| Omega-3 | Ghanaian | European American | -27.20 (-130.82, -113.22) | $1.0 \times 10^{-132}$ | -8.59 (-48.84, -30.67) | $2.0 \times 10^{-17}$ |
| | Ghanaian | African American | -27.51 (-140.40, -121.71) | $3.0 \times 10^{-135}$ | -11.92 (-65.67, -47.11) | $3.0 \times 10^{-31}$ |
| | African American | European American | 1.76 (-1.01, 19.07) | 0.0778 | 3.30 (6.74, 26.53) | 0.0010 |
| Omega-6 | Ghanaian | European American | 22.01 (339.34, 405.74) | $3.0x10^{-93}$ | 26.29 (469.05, 544.69) | $1.0 \times 10^{-124}$ |
| | Ghanaian | African American | 19.68 (318.47, 388.98) | $7.0 \times 10^{-77}$ | 21.00 (375.28, 452.60) | $1.0 \times 10^{-85}$ |
| | African American | European American | 0.97 (-19.04, 56.68) | 0.3298 | 4.42 (51.71, 134.14) | $1.0 \times 10^{-5}$ |
| Omega 6:3 | Ghanaian | European American | 41.07 (7.05, 7.75) | $1.0 \times 10^{-247}$ | 23.83 (4.87, 5.74) | $6.0 \times 10^{-106}$ |
| | Ghanaian | African American | 41.22 (7.51, 8.26) | $5.0 \times 10^{-249}$ | 24.32 (5.09, 5.98) | $1.0 \times 10^{-109}$ |
| | African American | European American | -2.37 (-0.89, -0.08) | 0.0177 | -0.95 (-0.71, 0.25) | 0.3414 |
| *trans* | Ghanaian | European American | 23.98 (25.83, 30.43) | $7.0 \times 10^{-108}$ | 21.90 (33.80, 40.45) | $7.0 \times 10^{-92}$ |
| | Ghanaian | African American | 17.13 (18.89, 23.78) | $3.0 \times 10^{-60}$ | 15.39 (23.27, 30.07) | $1.0 \times 10^{-49}$ |
| | African American | European American | 5.08 (4.17, 9.42) | $4.3 \times 10^{-7}$ | 5.66 (6.83, 14.07) | $1.9 \times 10^{-8}$ |

CI = Confidence Interval

* Bonferroni adjusted threshold for significance is $P = 0.0021$ following Student's t-test

All Student's t-tests were two-sided

**Supplementary Table 6.** Genotype frequencies for three SNPs in the *FADS1/2* locus among Ghanaian, African American and European American men (cases and controls combined)

| **Gene (SNP)** | **Ghanaian** *N(%)* | **African American** *N(%)* | **European American** *N(%)* |
|---|---|---|---|
| *FADS1* (rs174556) | | | |
| CC | 1170 (99.0) | 693 (86.4) | 434 (52.1) |
| CT | 12 (1.0) | 103 (12.8) | 333 (40.0) |
| TT | 0 (0) | 6 (0.8) | 66 (7.9) |
| *FADS2* (rs174577) | | | |
| CC | 464 (39.3) | 281 (40.6) | 341 (45.3) |
| CA | 552 (46.8) | 333 (48.1) | 328 (43.6) |
| AA | 164 (13.9) | 79 (11.4) | 84 (11.2) |
| *FADS2* (rs174583) | | | |
| CC | 686 (58.6) | 416 (52.7) | 373 (45.1) |
| CT | 412 (35.2) | 318 (40.3) | 362 (43.8) |
| TT | 72 (6.2) | 56 (7.1) | 92 (11.1) |

SNP = single nucleotide polymorphism

**Supplementary Table 7.** Fraction of variance for each of the fatty acids explained by SNP rs174556 among Ghanaian, African American, and European American controls

| | | Ghanaian | | | | African American | | | | European American | | | |
|---|---|---|---|---|---|---|---|---|---|---|---|---|---|
| **Fatty acids** | **Fatty acid classes** | ***N*** | **variance fraction (%)** | **F** | ***P**** | ***N*** | **variance fraction (%)** | **F** | ***P*** | ***N*** | **variance fraction (%)** | **F** | ***P*** |
| Arachidic | Saturated | 602 | 0.06 | 0.35 | 0.5547 | 350 | 0.1 | 0.17 | 0.8454 | 394 | 0.16 | 0.31 | 0.7323 |
| Behenic | Saturated | 602 | 0.01 | 0.07 | 0.7988 | 350 | 0.05 | 0.08 | 0.9247 | 394 | 0.93 | 1.83 | 0.1613 |
| Lignoceric | Saturated | 602 | 0.01 | 0.08 | 0.7714 | 350 | 0.15 | 0.26 | 0.7705 | 394 | 0.62 | 1.22 | 0.2974 |
| Myristic | Saturated | 602 | 0.02 | 0.11 | 0.7371 | 350 | 0.05 | 0.09 | 0.9152 | 394 | 0.18 | 0.35 | 0.7045 |
| Palmitic | Saturated | 602 | 0.00 | 0.02 | 0.8998 | 350 | 0.12 | 0.20 | 0.8190 | 394 | 0.43 | 0.84 | 0.4342 |
| Stearic | Saturated | 602 | 0.03 | 0.20 | 0.6578 | 350 | 0.05 | 0.08 | 0.9192 | 394 | 0.17 | 0.34 | 0.7112 |
| Eicosenoic | *cis* -Mono | 602 | 0.04 | 0.26 | 0.6090 | 350 | 0.51 | 0.89 | 0.4113 | 394 | 1.78 | 3.55 | 0.0296 |
| Nervonic | *cis* -Mono | 602 | 0.01 | 0.03 | 0.8525 | 350 | 1.11 | 1.95 | 0.1444 | 394 | 0.66 | 1.30 | 0.2744 |
| Oleic | *cis* -Mono | 602 | 0.05 | 0.28 | 0.5971 | 350 | 0.38 | 0.67 | 0.5136 | 394 | 0.73 | 1.45 | 0.2365 |
| Palmitoleic | *cis* -Mono | 602 | 0.02 | 0.14 | 0.7092 | 350 | 0.12 | 0.20 | 0.8171 | 394 | 1.54 | 3.05 | 0.0484 |
| alpha-Linolenic | Omega-3 | 602 | 0.73 | 4.43 | 0.0356 | 350 | 0.06 | 0.10 | 0.9057 | 394 | 0.76 | 1.50 | 0.2234 |
| Docosahexaenoic | Omega-3 | 602 | 0.01 | 0.08 | 0.7839 | 350 | 0.52 | 0.91 | 0.4039 | 394 | 0.22 | 0.44 | 0.6467 |
| Docosapentaenoic - n3 | Omega-3 | 602 | 0.01 | 0.08 | 0.7820 | 350 | 0.31 | 0.54 | 0.5827 | 394 | 0.72 | 1.43 | 0.2414 |
| Eicosapentaenoic | Omega-3 | 602 | 0.11 | 0.68 | 0.4089 | 350 | 0.2 | 0.34 | 0.7119 | 394 | 0.3 | 0.58 | 0.5605 |
| Arachidonic | Omega-6 | 602 | 0.40 | 2.44 | 0.1191 | 350 | 1.21 | 2.13 | 0.1209 | 394 | 14.34 | 32.73 | <0.0001 |
| Dihomo-g-linolenic | Omega-6 | 602 | 1.86 | 11.39 | 0.0008 | 350 | 2.18 | 3.87 | 0.0218 | 394 | 7.68 | 16.27 | <0.0001 |
| Docosapentaenoic - n6 | Omega-6 | 602 | 0.24 | 1.43 | 0.2329 | 350 | 0.02 | 0.03 | 0.9691 | 394 | 0.51 | 1.01 | 0.3666 |
| Docosatetraenoic | Omega-6 | 602 | 0.36 | 2.19 | 0.1392 | 350 | 0.19 | 0.33 | 0.7188 | 394 | 2.9 | 5.84 | 0.0032 |
| Eicosadienoic | Omega-6 | 602 | 0.39 | 2.34 | 0.1268 | 350 | 0.43 | 0.75 | 0.4747 | 394 | 1.41 | 2.80 | 0.0621 |
| gamma-Linolenic | Omega-6 | 602 | 0.08 | 0.46 | 0.4966 | 350 | 0.9 | 1.57 | 0.2094 | 394 | 6.64 | 13.90 | <0.0001 |
| Linoleic | Omega-6 | 602 | 0.21 | 1.27 | 0.2604 | 350 | 0.6 | 1.05 | 0.3526 | 394 | 0.07 | 0.14 | 0.8712 |
| Elaidic | *trans* | 602 | 0.12 | 0.73 | 0.3940 | 350 | 1.43 | 2.52 | 0.0816 | 394 | 0.06 | 0.12 | 0.8874 |
| Palmitelaidic | *trans* | 602 | 0.20 | 1.21 | 0.2719 | 350 | 3.01 | 5.39 | 0.0050 | 394 | 0.12 | 0.23 | 0.7915 |
| Linoelaidic | *trans* | 602 | 0.01 | 0.05 | 0.8307 | 350 | 0.28 | 0.48 | 0.6174 | 394 | 0.05 | 0.11 | 0.9003 |

Analysis of variance (ANOVA) was performed for the levels of each of the 24 fatty aids as a function of rs174556 in the *FADS1* gene among men without prostate cancer.
*Bonferroni adjusted threshold for significance is $P = 0.0021$

**Supplementary Table 8.** Fraction of variance for each of the fatty acids explained by SNP rs174556 among Ghanaian, African American, and European American cases

| Fatty acids | Fatty acid classes | Ghanaian | | | | African American | | | | European American | | | |
|---|---|---|---|---|---|---|---|---|---|---|---|---|---|
| | | ***N*** | **variance fraction (%)** | **F** | ***P*** | ***N*** | **variance fraction (%)** | **F** | ***P*** | ***N*** | **variance fraction (%)** | **F** | ***P*** |
| Arachidic | Saturated | 511 | 0.09 | 0.46 | 0.4993 | 344 | 0.04 | 0.08 | 0.9273 | 362 | 1.14 | 2.07 | 0.1275 |
| Behenic | Saturated | 511 | 0.10 | 0.52 | 0.4692 | 344 | 0.33 | 0.56 | 0.5694 | 362 | 0.5 | 0.90 | 0.4062 |
| Lignoceric | Saturated | 511 | 0.08 | 0.40 | 0.5258 | 344 | 0.17 | 0.28 | 0.7534 | 362 | 0.35 | 0.62 | 0.5368 |
| Myristic | Saturated | 511 | 0.19 | 0.95 | 0.3292 | 344 | 0.41 | 0.71 | 0.4925 | 362 | 0.1 | 0.18 | 0.8388 |
| Palmitic | Saturated | 511 | 0.17 | 0.86 | 0.3551 | 344 | 0.4 | 0.69 | 0.5022 | 362 | 0.05 | 0.09 | 0.9183 |
| Stearic | Saturated | 511 | 0.17 | 0.87 | 0.3526 | 344 | 0.36 | 0.61 | 0.5451 | 362 | 0.25 | 0.44 | 0.6428 |
| Eicosenoic | *cis* -Mono | 511 | 0.24 | 1.21 | 0.2717 | 344 | 0.32 | 0.55 | 0.5792 | 362 | 0.55 | 0.99 | 0.3724 |
| Nervonic | *cis* -Mono | 511 | 0.09 | 0.44 | 0.5057 | 344 | 0.45 | 0.77 | 0.4643 | 362 | 0.45 | 0.81 | 0.4458 |
| Oleic | *cis* -Mono | 511 | 0.13 | 0.65 | 0.4200 | 344 | 0.35 | 0.60 | 0.5517 | 362 | 0.28 | 0.50 | 0.6075 |
| Palmitoleic | *cis* -Mono | 511 | 0.01 | 0.07 | 0.7930 | 344 | 0.42 | 0.72 | 0.4873 | 362 | 0.08 | 0.15 | 0.8603 |
| alpha-Linolenic | Omega-3 | 511 | 0.01 | 0.03 | 0.8596 | 344 | 0.33 | 0.57 | 0.5658 | 362 | 1.2 | 2.17 | 0.1154 |
| Docosahexaenoic | Omega-3 | 511 | 0.03 | 0.14 | 0.7111 | 344 | 0.92 | 1.59 | 0.2059 | 362 | 0.19 | 0.33 | 0.7163 |
| Docosapentaenoic - n3 | Omega-3 | 511 | 0.02 | 0.09 | 0.7696 | 344 | 0.84 | 1.45 | 0.2357 | 362 | 1.27 | 2.30 | 0.1016 |
| Eicosapentaenoic | Omega-3 | 511 | 0.06 | 0.28 | 0.5962 | 344 | 0.39 | 0.66 | 0.5158 | 362 | 0.58 | 1.06 | 0.3490 |
| Arachidonic | Omega-6 | 511 | 0.07 | 0.38 | 0.5378 | 344 | 4.74 | 8.48 | 0.0003 | 362 | 14.02 | 29.27 | <0.0001 |
| Dihomo-g-linolenic | Omega-6 | 511 | 0.06 | 0.33 | 0.5684 | 344 | 1.48 | 2.57 | 0.0781 | 362 | 3.77 | 7.04 | 0.0010 |
| Docosapentaenoic - n6 | Omega-6 | 511 | 0.06 | 0.29 | 0.5925 | 344 | 0.36 | 0.62 | 0.5378 | 362 | 0.65 | 1.18 | 0.3100 |
| Docosatetraenoic | Omega-6 | 511 | 0.15 | 0.75 | 0.3859 | 344 | 0.69 | 1.18 | 0.3073 | 362 | 3.25 | 6.02 | 0.0027 |
| Eicosadienoic | Omega-6 | 511 | 0.19 | 0.94 | 0.3317 | 344 | 0.22 | 0.37 | 0.6915 | 362 | 0.68 | 1.23 | 0.2928 |
| gamma-Linolenic | Omega-6 | 511 | 0.07 | 0.38 | 0.5404 | 344 | 1.46 | 2.52 | 0.0819 | 362 | 6.47 | 12.42 | <0.0001 |
| Linoleic | Omega-6 | 511 | 0.11 | 0.55 | 0.4570 | 344 | 0.14 | 0.24 | 0.7889 | 362 | 0.77 | 1.40 | 0.2487 |
| Elaidic | *trans* | 511 | 0.22 | 1.14 | 0.2862 | 344 | 1.03 | 1.78 | 0.1707 | 362 | 0.79 | 1.43 | 0.2412 |
| Palmitelaidic | *trans* | 511 | 0.07 | 0.37 | 0.5408 | 344 | 1.87 | 3.26 | 0.0397 | 362 | 0.23 | 0.41 | 0.6634 |
| Linoelaidic | *trans* | 511 | 0.31 | 1.60 | 0.2066 | 344 | 1.36 | 2.35 | 0.0967 | 362 | 0.07 | 0.12 | 0.8847 |

Analysis of variance (ANOVA) was performed for the levels of each of the 24 fatty aids as a function of genetic variation in the *FADS2* gene (rs174556) among men with prostate cancer.

*Bonferroni adjusted threshold for significance is $P = 0.0021$

**Supplementary Table 9.** Fraction of variance for each of the fatty acids explained by SNP rs174583 among Ghanaian, African American, and European American controls

| Fatty acids | Fatty acid classes | Ghanaian *N* | Ghanaian variance fraction (%) | Ghanaian F | Ghanaian *P** | African American *N* | African American variance fraction (%) | African American F | African American *P* | European American *N* | European American variance fraction (%) | European American F | European American *P* |
|---|---|---|---|---|---|---|---|---|---|---|---|---|---|
| Arachidic | Saturated | 593 | 0.08 | 0.22 | 0.7989 | 347 | 0.27 | 0.47 | 0.6258 | 391 | 0.33 | 0.63 | 0.5317 |
| Behenic | Saturated | 593 | 0.18 | 0.54 | 0.5853 | 347 | 0.66 | 1.14 | 0.3218 | 391 | 0.97 | 1.89 | 0.1522 |
| Lignoceric | Saturated | 593 | 0.13 | 0.38 | 0.6821 | 347 | 0.56 | 0.96 | 0.3826 | 391 | 0.6 | 1.16 | 0.3138 |
| Myristic | Saturated | 593 | 0.41 | 1.22 | 0.2960 | 347 | 0.26 | 0.46 | 0.6337 | 391 | 0.09 | 0.17 | 0.8400 |
| Palmitic | Saturated | 593 | 0.03 | 0.09 | 0.9126 | 347 | 0.06 | 0.11 | 0.8943 | 391 | 0.09 | 0.17 | 0.8397 |
| Stearic | Saturated | 593 | 0.03 | 0.08 | 0.9241 | 347 | 0.11 | 0.18 | 0.8328 | 391 | 0.22 | 0.43 | 0.6480 |
| Eicosenoic | *cis* -Mono | 593 | 0.10 | 0.30 | 0.7445 | 347 | 0.52 | 0.89 | 0.4104 | 391 | 1.4 | 2.76 | 0.0646 |
| Nervonic | *cis* -Mono | 593 | 0.57 | 1.68 | 0.1877 | 347 | 0.15 | 0.25 | 0.7752 | 391 | 0 | 0.00 | 0.9966 |
| Oleic | *cis* -Mono | 593 | 0.12 | 0.34 | 0.7121 | 347 | 0.2 | 0.34 | 0.7125 | 391 | 0.13 | 0.25 | 0.7797 |
| Palmitoleic | *cis* -Mono | 593 | 0.52 | 1.54 | 0.2149 | 347 | 0.02 | 0.04 | 0.9589 | 391 | 0.26 | 0.51 | 0.6028 |
| alpha-Linolenic | Omega-3 | 593 | 0.04 | 0.13 | 0.8822 | 347 | 3.79 | 6.77 | 0.0013 | 391 | 0.08 | 0.16 | 0.8550 |
| Docosahexaenoic | Omega-3 | 593 | 0.50 | 1.48 | 0.2292 | 347 | 0.38 | 0.65 | 0.5236 | 391 | 0.06 | 0.12 | 0.8914 |
| Docosapentaenoic - n3 | Omega-3 | 593 | 0.32 | 0.95 | 0.3872 | 347 | 0.22 | 0.38 | 0.6809 | 391 | 0.89 | 1.75 | 0.1749 |
| Eicosapentaenoic | Omega-3 | 593 | 0.21 | 0.61 | 0.5412 | 347 | 2.08 | 3.64 | 0.0271 | 391 | 0.11 | 0.22 | 0.8057 |
| Arachidonic | Omega-6 | 593 | 0.19 | 0.56 | 0.5723 | 347 | 1.18 | 2.06 | 0.1290 | 391 | 19.02 | 45.57 | <0.0001 |
| Dihomo-g-linolenic | Omega-6 | 593 | 0.17 | 0.50 | 0.6068 | 347 | 0.45 | 0.78 | 0.4609 | 391 | 4.39 | 8.92 | 0.0002 |
| Docosapentaenoic - n6 | Omega-6 | 593 | 0.51 | 1.52 | 0.2195 | 347 | 0.35 | 0.61 | 0.5453 | 391 | 1.29 | 2.53 | 0.0806 |
| Docosatetraenoic | Omega-6 | 593 | 0.10 | 0.28 | 0.7530 | 347 | 0.28 | 0.48 | 0.6173 | 391 | 3.86 | 7.78 | 0.0005 |
| Eicosadienoic | Omega-6 | 593 | 0.03 | 0.09 | 0.9171 | 347 | 0.21 | 0.37 | 0.6922 | 391 | 0.53 | 1.03 | 0.3581 |
| gamma-Linolenic | Omega-6 | 593 | 0.22 | 0.64 | 0.5293 | 347 | 0.3 | 0.52 | 0.5928 | 391 | 9.77 | 21.00 | <0.0001 |
| Linoleic | Omega-6 | 593 | 0.62 | 1.84 | 0.1596 | 347 | 0.22 | 0.37 | 0.6897 | 391 | 0.02 | 0.05 | 0.9549 |
| Elaidic | *trans* | 593 | 0.09 | 0.26 | 0.7704 | 347 | 0.14 | 0.25 | 0.7807 | 391 | 0.47 | 0.91 | 0.4025 |
| Palmitelaidic | *trans* | 593 | 0.12 | 0.35 | 0.7068 | 347 | 0.11 | 0.19 | 0.8266 | 391 | 0.08 | 0.16 | 0.8561 |
| Linoelaidic | *trans* | 593 | 0.09 | 0.26 | 0.7675 | 347 | 0.34 | 0.58 | 0.5610 | 391 | 0.27 | 0.53 | 0.5862 |

Analysis of variance (ANOVA) was performed for the levels of each of the 24 fatty aids as a function of genetic variation in the *FADS2* gene (rs174583) among men without prostate cancer.

*Bonferroni adjusted threshold for significance is $P = 0.0021$

**Supplementary Table 10.** Fraction of variance for each of the fatty acids explained by rs174583 in Ghanaian, African American, and European American cases

| **Fatty acids** | **Fatty acid classes** | **Ghanaian** | | | | **African American** | | | | **European American** | | | |
|---|---|---|---|---|---|---|---|---|---|---|---|---|---|
| | | ***N*** | **variance fraction (%)** | **F** | ***P**** | ***N*** | **variance fraction (%)** | **F** | ***P*** | ***N*** | **variance fraction (%)** | **F** | ***P*** |
| Arachidic | Saturated | 509 | 0.23 | 0.57 | 0.5649 | 340 | 0.75 | 1.27 | 0.2829 | 361 | 0.51 | 0.91 | 0.4039 |
| Behenic | Saturated | 509 | 0.05 | 0.14 | 0.8701 | 340 | 0.2 | 0.35 | 0.7084 | 361 | 0.19 | 0.34 | 0.7119 |
| Lignoceric | Saturated | 509 | 0.09 | 0.22 | 0.8021 | 340 | 0.1 | 0.17 | 0.8455 | 361 | 0.25 | 0.45 | 0.6397 |
| Myristic | Saturated | 509 | 0.17 | 0.44 | 0.6469 | 340 | 1.36 | 2.31 | 0.1004 | 361 | 0.06 | 0.11 | 0.8987 |
| Palmitic | Saturated | 509 | 0.14 | 0.36 | 0.6952 | 340 | 0.73 | 1.23 | 0.2923 | 361 | 0.01 | 0.02 | 0.9845 |
| Stearic | Saturated | 509 | 0.49 | 1.24 | 0.2891 | 340 | 0.73 | 1.24 | 0.2914 | 361 | 0.06 | 0.10 | 0.9030 |
| Eicosenoic | *cis* -Mono | 509 | 0.39 | 0.99 | 0.3739 | 340 | 1.89 | 3.25 | 0.0398 | 361 | 0.49 | 0.88 | 0.4155 |
| Nervonic | *cis* -Mono | 509 | 0.07 | 0.18 | 0.8342 | 340 | 0.61 | 1.03 | 0.3591 | 361 | 0.29 | 0.51 | 0.5988 |
| Oleic | *cis* -Mono | 509 | 0.61 | 1.56 | 0.2108 | 340 | 1.26 | 2.16 | 0.1175 | 361 | 0.19 | 0.34 | 0.7124 |
| Palmitoleic | *cis* -Mono | 509 | 0.31 | 0.79 | 0.4533 | 340 | 0.48 | 0.81 | 0.4472 | 361 | 0.18 | 0.32 | 0.7249 |
| alpha-Linolenic | Omega-3 | 509 | 0.54 | 1.37 | 0.2544 | 340 | 1.01 | 1.73 | 0.1795 | 361 | 0.7 | 1.26 | 0.2844 |
| Docosahexaenoic | Omega-3 | 509 | 0.02 | 0.04 | 0.9621 | 340 | 0.25 | 0.42 | 0.6576 | 361 | 0.48 | 0.87 | 0.4210 |
| Docosapentaenoic - n3 | Omega-3 | 509 | 0.02 | 0.06 | 0.9438 | 340 | 0.06 | 0.11 | 0.8979 | 361 | 1.72 | 3.14 | 0.0447 |
| Eicosapentaenoic | Omega-3 | 509 | 0.11 | 0.28 | 0.7547 | 340 | 0.02 | 0.03 | 0.9699 | 361 | 0.94 | 1.70 | 0.1847 |
| Arachidonic | Omega-6 | 509 | 0.05 | 0.13 | 0.8756 | 340 | 1.22 | 2.08 | 0.1261 | 361 | 16.86 | 36.31 | <0.0001 |
| Dihomo-g-linolenic | Omega-6 | 509 | 0.22 | 0.55 | 0.5747 | 340 | 0.64 | 1.09 | 0.3369 | 361 | 3.39 | 6.28 | 0.0021 |
| Docosapentaenoic - n6 | Omega-6 | 509 | 0.01 | 0.02 | 0.9842 | 340 | 0.42 | 0.70 | 0.4954 | 361 | 1.21 | 2.19 | 0.1135 |
| Docosatetraenoic | Omega-6 | 509 | 0.07 | 0.17 | 0.8436 | 340 | 0.14 | 0.24 | 0.7869 | 361 | 3.58 | 6.64 | 0.0015 |
| Eicosadienoic | Omega-6 | 509 | 0.04 | 0.11 | 0.8977 | 340 | 1.54 | 2.63 | 0.0737 | 361 | 0.61 | 1.10 | 0.3324 |
| gamma-Linolenic | Omega-6 | 509 | 0.15 | 0.39 | 0.6768 | 340 | 1.18 | 2.00 | 0.1364 | 361 | 7.11 | 13.70 | <0.0001 |
| Linoleic | Omega-6 | 509 | 0.26 | 0.67 | 0.5115 | 340 | 0.98 | 1.66 | 0.1910 | 361 | 0.26 | 0.46 | 0.6304 |
| Elaidic | *trans* | 509 | 1.17 | 2.98 | 0.0515 | 340 | 0.17 | 0.29 | 0.7474 | 361 | 0.17 | 0.30 | 0.7427 |
| Palmitelaidic | *trans* | 509 | 0.02 | 0.06 | 0.9405 | 340 | 0.55 | 0.93 | 0.3960 | 361 | 0.23 | 0.41 | 0.6640 |
| Linoelaidic | *trans* | 509 | 0.06 | 0.16 | 0.8533 | 340 | 0.56 | 0.95 | 0.3864 | 361 | 0.04 | 0.07 | 0.9361 |

Analysis of variance (ANOVA) was performed for the levels of each of the 24 fatty aids as a function of genetic variation in the *FADS2* gene (rs174583) among men with prostate cancer.

*Bonferroni adjusted threshold for significance is $P = 0.0021$

**Supplementary Table 11.** Fraction of variance for each of the fatty acids explained by rs174577 in Ghanaian, African American, and European American controls

| Fatty acids | Fatty acid classes | Ghanaian | | | | African American | | | | European American | | | |
|---|---|---|---|---|---|---|---|---|---|---|---|---|---|
| | | *N* | variance fraction (%) | F | *P** | *N* | variance fraction (%) | F | *P* | *N* | variance fraction (%) | F | *P* |
| Arachidic | Saturated | 600 | 0.22 | 0.67 | 0.5115 | 349 | 0.86 | 1.50 | 0.2238 | 392 | 0.22 | 0.43 | 0.6538 |
| Behenic | Saturated | 600 | 0.35 | 1.04 | 0.3526 | 349 | 0.65 | 1.12 | 0.3260 | 392 | 0.79 | 1.56 | 0.2123 |
| Lignoceric | Saturated | 600 | 0.36 | 1.09 | 0.3369 | 349 | 0.56 | 0.98 | 0.3755 | 392 | 0.43 | 0.84 | 0.4323 |
| Myristic | Saturated | 600 | 0.05 | 0.14 | 0.8693 | 349 | 0.2 | 0.35 | 0.7071 | 392 | 0.08 | 0.16 | 0.8552 |
| Palmitic | Saturated | 600 | 0.19 | 0.57 | 0.5650 | 349 | 0.23 | 0.39 | 0.6766 | 392 | 0.1 | 0.20 | 0.8222 |
| Stearic | Saturated | 600 | 0.04 | 0.12 | 0.8850 | 349 | 0.42 | 0.74 | 0.4792 | 392 | 0.12 | 0.23 | 0.7944 |
| Eicosenoic | *cis* -Mono | 600 | 0.11 | 0.33 | 0.7225 | 349 | 1.12 | 1.96 | 0.1431 | 392 | 1.56 | 3.09 | 0.0468 |
| Nervonic | *cis* -Mono | 600 | 0.38 | 1.13 | 0.3223 | 349 | 0.38 | 0.67 | 0.5148 | 392 | 0 | 0.00 | 0.9986 |
| Oleic | *cis* -Mono | 600 | 0.23 | 0.69 | 0.5037 | 349 | 0.61 | 1.05 | 0.3500 | 392 | 0.18 | 0.35 | 0.7017 |
| Palmitoleic | *cis* -Mono | 600 | 0.53 | 1.59 | 0.2054 | 349 | 0.07 | 0.11 | 0.8924 | 392 | 0.35 | 0.68 | 0.5069 |
| alpha-Linolenic | Omega-3 | 600 | 0.05 | 0.15 | 0.8640 | 349 | 1.86 | 3.29 | 0.0385 | 392 | 0.17 | 0.34 | 0.7145 |
| Docosahexaenoic | Omega-3 | 600 | 0.12 | 0.35 | 0.7046 | 349 | 0.66 | 1.15 | 0.3180 | 392 | 0.02 | 0.04 | 0.9569 |
| Docosapentaenoic - n3 | Omega-3 | 600 | 0.05 | 0.15 | 0.8597 | 349 | 0.13 | 0.23 | 0.7927 | 392 | 0.73 | 1.42 | 0.2424 |
| Eicosapentaenoic | Omega-3 | 600 | 0.37 | 1.10 | 0.3335 | 349 | 1.98 | 3.50 | 0.0312 | 392 | 0.3 | 0.59 | 0.5563 |
| Arachidonic | Omega-6 | 600 | 0.64 | 1.91 | 0.1485 | 349 | 1.13 | 1.98 | 0.1395 | 392 | 18.85 | 45.19 | <0.0001 |
| Dihomo-g-linolenic | Omega-6 | 600 | 0.02 | 0.05 | 0.9472 | 349 | 0.3 | 0.52 | 0.5971 | 392 | 4.54 | 9.24 | 0.0001 |
| Docosapentaenoic - n6 | Omega-6 | 600 | 0.28 | 0.84 | 0.4322 | 349 | 0.22 | 0.39 | 0.6774 | 392 | 1.28 | 2.52 | 0.0815 |
| Docosatetraenoic | Omega-6 | 600 | 0.08 | 0.22 | 0.7987 | 349 | 0.31 | 0.54 | 0.5825 | 392 | 3.98 | 8.07 | 0.0004 |
| Eicosadienoic | Omega-6 | 600 | 0.26 | 0.78 | 0.4608 | 349 | 0.3 | 0.51 | 0.5991 | 392 | 0.75 | 1.47 | 0.2311 |
| gamma-Linolenic | Omega-6 | 600 | 0.17 | 0.52 | 0.5943 | 349 | 0.56 | 0.98 | 0.3762 | 392 | 9.47 | 20.33 | <0.0001 |
| Linoleic | Omega-6 | 600 | 0.18 | 0.53 | 0.5916 | 349 | 0.01 | 0.02 | 0.9774 | 392 | 0.07 | 0.14 | 0.8712 |
| Elaidic | *trans* | 600 | 0.05 | 0.16 | 0.8496 | 349 | 0.05 | 0.08 | 0.9247 | 392 | 0.3 | 0.59 | 0.5564 |
| Palmitelaidic | *trans* | 600 | 0.11 | 0.32 | 0.7273 | 349 | 0.22 | 0.39 | 0.6773 | 392 | 0.01 | 0.03 | 0.9714 |
| Linoelaidic | *trans* | 600 | 0.00 | 0.01 | 0.9912 | 349 | 0.15 | 0.25 | 0.7758 | 392 | 0.17 | 0.33 | 0.7183 |

Analysis of variance (ANOVA) was performed for the levels of each of the 24 fatty aids as a function of genetic variation in the *FADS2* gene (rs174577) among men without prostate cancer.

*Bonferroni adjusted threshold for significance is $P = 0.0021$

**Supplementary Table 12.** Fraction of variance for each of the fatty acids explained by rs174577 in Ghanaian, African American, and European American cases

| Fatty acids | Fatty acid classes | Ghanaian | | | | African American | | | | European American | | | |
|---|---|---|---|---|---|---|---|---|---|---|---|---|---|
| | | ***N*** | **variance fraction (%)** | **F** | ***P*** | ***N*** | **variance fraction (%)** | **F** | ***P*** | ***N*** | **variance fraction (%)** | **F** | ***P*** |
| Arachidic | Saturated | 511 | 0.26 | 0.66 | 0.5193 | 344 | 2.19 | 3.83 | 0.0228 | 361 | 0.77 | 1.39 | 0.2507 |
| Behenic | Saturated | 511 | 0.02 | 0.06 | 0.9402 | 344 | 0.24 | 0.42 | 0.6601 | 361 | 0.3 | 0.54 | 0.5817 |
| Lignoceric | Saturated | 511 | 0.11 | 0.28 | 0.7557 | 344 | 0.23 | 0.40 | 0.6707 | 361 | 0.22 | 0.40 | 0.6701 |
| Myristic | Saturated | 511 | 0.14 | 0.36 | 0.6949 | 344 | 2.25 | 3.92 | 0.0207 | 361 | 0.1 | 0.19 | 0.83 |
| Palmitic | Saturated | 511 | 0.05 | 0.12 | 0.8835 | 344 | 2.89 | 5.08 | 0.0067 | 361 | 0 | 0.01 | 0.9917 |
| Stearic | Saturated | 511 | 0.10 | 0.26 | 0.7709 | 344 | 2.92 | 5.13 | 0.0064 | 361 | 0.05 | 0.08 | 0.9197 |
| Eicosenoic | *cis* -Mono | 511 | 0.42 | 1.07 | 0.3423 | 344 | 5.13 | 9.23 | 0.0001 | 361 | 0.52 | 0.94 | 0.3917 |
| Nervonic | *cis* -Mono | 511 | 0.53 | 1.36 | 0.2581 | 344 | 0.61 | 1.05 | 0.3497 | 361 | 0.26 | 0.46 | 0.6312 |
| Oleic | *cis* -Mono | 511 | 0.03 | 0.07 | 0.9295 | 344 | 4.58 | 8.19 | 0.0003 | 361 | 0.17 | 0.30 | 0.7378 |
| Palmitoleic | *cis* -Mono | 511 | 0.23 | 0.59 | 0.5529 | 344 | 2.31 | 4.04 | 0.0185 | 361 | 0.13 | 0.23 | 0.7908 |
| alpha-Linolenic | Omega-3 | 511 | 0.02 | 0.06 | 0.9432 | 344 | 2.56 | 4.47 | 0.0121 | 361 | 0.68 | 1.23 | 0.2938 |
| Docosahexaenoic | Omega-3 | 511 | 0.32 | 0.83 | 0.4386 | 344 | 1.88 | 3.27 | 0.0393 | 361 | 0.45 | 0.82 | 0.4427 |
| Docosapentaenoic - n3 | Omega-3 | 511 | 0.10 | 0.25 | 0.7824 | 344 | 2.53 | 4.42 | 0.0127 | 361 | 1.97 | 3.59 | 0.0285 |
| Eicosapentaenoic | Omega-3 | 511 | 0.10 | 0.25 | 0.7817 | 344 | 0.73 | 1.25 | 0.2869 | 361 | 0.94 | 1.70 | 0.1850 |
| Arachidonic | Omega-6 | 511 | 0.04 | 0.11 | 0.8932 | 344 | 0.16 | 0.27 | 0.7641 | 361 | 18.16 | 39.73 | <0.0001 |
| Dihomo-g-linolenic | Omega-6 | 511 | 0.30 | 0.77 | 0.4646 | 344 | 1.86 | 3.23 | 0.0409 | 361 | 3.2 | 5.91 | 0.0030 |
| Docosapentaenoic - n6 | Omega-6 | 511 | 0.08 | 0.21 | 0.8135 | 344 | 0.51 | 0.87 | 0.4199 | 361 | 1.22 | 2.22 | 0.1102 |
| Docosatetraenoic | Omega-6 | 511 | 0.20 | 0.52 | 0.5948 | 344 | 0.67 | 1.16 | 0.3159 | 361 | 4.32 | 8.08 | 0.0004 |
| Eicosadienoic | Omega-6 | 511 | 0.11 | 0.28 | 0.7554 | 344 | 3.14 | 5.53 | 0.0043 | 361 | 0.55 | 0.99 | 0.3717 |
| gamma-Linolenic | Omega-6 | 511 | 0.07 | 0.17 | 0.8475 | 344 | 0.04 | 0.08 | 0.9274 | 361 | 7.91 | 15.37 | <0.0001 |
| Linoleic | Omega-6 | 511 | 0.35 | 0.89 | 0.4115 | 344 | 1.72 | 2.98 | 0.0521 | 361 | 0.26 | 0.47 | 0.6272 |
| Elaidic | *trans* | 511 | 0.51 | 1.31 | 0.2719 | 344 | 0.67 | 1.15 | 0.3194 | 361 | 0.63 | 1.13 | 0.3238 |
| Palmitelaidic | *trans* | 511 | 0.16 | 0.40 | 0.6712 | 344 | 2.55 | 4.46 | 0.0122 | 361 | 0.11 | 0.19 | 0.8244 |
| Linoelaidic | *trans* | 511 | 0.02 | 0.05 | 0.9554 | 344 | 1.25 | 2.16 | 0.1165 | 361 | 0.03 | 0.05 | 0.9519 |

Analysis of variance (ANOVA) was performed for the levels of each of the 24 fatty aids as a function of genetic variation in the *FADS2* gene (rs174577) among men with prostate cancer.

*Bonferroni adjusted threshold for significance is $P = 0.0021$

**Supplementary Table 13.** Association between fatty acid classes and prostate cancer among Ghanaian, African American, and European American men

| Fatty Acid Class | All | | | | Ghanaian | | | | African American | | | | European American | | | |
|---|---|---|---|---|---|---|---|---|---|---|---|---|---|---|---|---|
| | Control *N* | Case *N* | OR (95% CI)[a] | *P*-value* | Control *N* | Case *N* | OR (95% CI)[b] | *P*-value | Control *N* | Case *N* | OR (95% CI)[b] | *P*-value | Control *N* | Case *N* | OR (95% CI)[b] | *P*-value |
| **Saturated** | | | | | | | | | | | | | | | | |
| Low | 751 | 624 | Ref | | 329 | 243 | Ref | | 222 | 221 | Ref | | 200 | 160 | Ref | |
| High | 752 | 807 | 1.34 (1.15-1.57) | $2.1 \times 10^{-4}$ | 329 | 342 | 1.30 (0.94-1.80) | 0.113 | 159 | 186 | 1.16 (0.86-1.56) | 0.340 | 264 | 279 | 1.22 (0.92-1.60) | 0.163 |
| Continuous | 1503 | 1431 | 1.69 (1.40-2.05) | $1.7 \times 10^{-7}$ | 658 | 585 | 1.89 (1.21-2.96) | 0.005 | 381 | 407 | 1.19 (0.84-1.70) | 0.323 | 464 | 439 | 1.53 (1.12-2.10) | 0.008 |
| ***cis-* Mono** | | | | | | | | | | | | | | | | |
| Low | 752 | 624 | Ref | | 329 | 250 | Ref | | 228 | 223 | Ref | | 195 | 151 | Ref | |
| High | 751 | 807 | 1.36 (1.17-1.59) | $9.0 \times 10^{-5}$ | 329 | 335 | 1.58 (1.14-2.18) | 0.006 | 153 | 184 | 1.17 (0.87-1.58) | 0.291 | 269 | 288 | 1.29 (0.98-1.70) | 0.073 |
| Continuous | 1503 | 1431 | 1.40 (1.20-1.64) | $3.8 \times 10^{-5}$ | 658 | 585 | 1.69 (1.16-2.44) | 0.006 | 381 | 407 | 1.09 (0.82-1.45) | 0.554 | 464 | 439 | 1.32 (1.02-1.72) | 0.036 |
| **Omega-3** | | | | | | | | | | | | | | | | |
| Low | 752 | 879 | Ref | | 329 | 439 | Ref | | 206 | 228 | Ref | | 217 | 212 | Ref | |
| High | 751 | 552 | 0.66 (0.56-0.77) | $2.4 \times 10^{-7}$ | 329 | 146 | 0.31 (0.22-0.45) | $7.8 \times 10^{-11}$ | 175 | 179 | 1.03 (0.77-1.39) | 0.829 | 247 | 227 | 1.01 (0.77-1.31) | 0.970 |
| Continuous | 1503 | 1431 | 0.54 (0.48-0.61) | $8.1 \times 10^{-24}$ | 658 | 585 | 0.21 (0.15-0.28) | $3.2 \times 10^{-26}$ | 381 | 407 | 1.04 (0.77-1.39) | 0.811 | 464 | 439 | 1.08 (0.86-1.36) | 0.516 |
| **Omega-6** | | | | | | | | | | | | | | | | |
| Low | 752 | 794 | Ref | | 329 | 377 | Ref | | 197 | 219 | Ref | | 226 | 198 | Ref | |
| High | 751 | 637 | 0.88 (0.75-1.03) | 0.118 | 329 | 208 | 0.52 (0.37-0.73) | $1.5 \times 10^{-4}$ | 184 | 188 | 0.89 (0.66-1.20) | 0.451 | 238 | 241 | 1.07 (0.82-1.41) | 0.616 |
| Continuous | 1503 | 1431 | 0.84 (0.68-1.04) | 0.116 | 658 | 585 | 0.24 (0.15-0.37) | $3.8 \times 10^{-10}$ | 381 | 407 | 0.86 (0.54-1.35) | 0.510 | 464 | 439 | 1.51 (0.99-2.30) | 0.057 |
| **Omega 6:3** | | | | | | | | | | | | | | | | |
| Low | 749 | 513 | Ref | | 326 | 120 | Ref | | 184 | 182 | Ref | | 239 | 211 | Ref | |
| High | 754 | 918 | 1.75 (1.50-2.06) | $4.2 \times 10^{-12}$ | 332 | 465 | 3.20 (2.25-4.55) | $1.5 \times 10^{-11}$ | 197 | 225 | 0.97 (0.72-1.32) | 0.866 | 225 | 228 | 0.99 (0.75-1.30) | 0.918 |
| Continuous | 1503 | 1431 | 1.96 (1.72-2.24) | $1.4 \times 10^{-23}$ | 658 | 585 | 4.23 (3.12-5.73) | $3.4 \times 10^{-21}$ | 381 | 407 | 0.86 (0.60-1.23) | 0.411 | 464 | 439 | 1.07 (0.81-1.41) | 0.623 |
| ***trans*** | | | | | | | | | | | | | | | | |
| Low | 752 | 497 | Ref | | 329 | 194 | Ref | | 237 | 176 | Ref | | 186 | 127 | Ref | |
| High | 751 | 934 | 1.94 (1.65-2.28) | $3.0 \times 10^{-16}$ | 329 | 391 | 1.82 (1.31-2.54) | $3.7 \times 10^{-4}$ | 144 | 231 | 2.23 (1.65-3.01) | $2.2 \times 10^{-7}$ | 278 | 312 | 1.56 (1.17-2.07) | 0.002 |
| Continuous | 1503 | 1431 | 1.80 (1.60-2.03) | $4.8 \times 10^{-21}$ | 658 | 585 | 2.12 (1.52-2.98) | $1.5 \times 10^{-5}$ | 381 | 407 | 1.80 (1.47-2.22) | $1.0 \times 10^{-7}$ | 464 | 439 | 1.55 (1.29-1.86) | $1.6 \times 10^{-6}$ |

[a]Logistic regression comparing high vs. low (ref.) fatty acid serum concentrations or with continuous data adjusted for age at recruitment, BMI, education, smoking history, diabetes, aspirin use, and population group

[b]Logistic regression adjusted for age at recruitment, BMI, education, smoking history, diabetes, and aspirin use

High fatty acid classes defined by the median score in the control population (≤ median vs. > median) as listed in Supp. Table 14

OR = Odds Ratio, CI = Confidence Interval

*Bonferroni adjusted threshold for significance is $P = 0.007$

**Supplementary Table 14**. Median cutoffs within the control population for each fatty acid class in the NCI-Maryland and NCI-Ghana cohorts

| Fatty Acid Class | NCI-MD | NCI-Ghana |
|---|---|---|
| **Saturated** | | |
| Median | 928.71 | 916.26 |
| ***cis-* Mono** | | |
| Median | 673.50 | 682.08 |
| **Omega-3** | | |
| Median | 110.24 | 235.11 |
| **Omega-6** | | |
| Median | 1267.62 | 917.85 |
| **Omega 6:3** | | |
| Median | 11.76 | 3.90 |
| ***trans*** | | |
| Median | 31.35 | 11.71 |
| **Elaidic Acid** | | |
| Median | 17.38 | 4.06 |
| **Palmitelaidic Acid** | | |
| Median | 4.48 | 2.70 |
| **Linoelaidic Acid** | | |
| Median | 9.03 | 4.67 |

**Supplementary Table 15.** Association of individual fatty acids with National Comprehensive Cancer Network (NCCN) risk scores for prostate cancer aggressiveness in the NCI-Maryland cohort

| | | NCCN Risk Score (4 categories) | | | | |
|---|---|---|---|---|---|---|
| | | **Low** | **Intermediate** | **High/Very High** | **Regional/Metastatic** | |
| **Fatty Acid** | **Fatty acid classes** | | **OR (95% CI)*** | **OR (95% CI)** | **OR (95% CI)** | ***P* trend** |
| Arachidic | Saturated | Ref | 1.01 (0.70, 1.47) | 1.04 (0.68, 1.60) | 1.37 (0.69, 2.72) | 0.501 |
| Behenic | Saturated | Ref | 0.79 (0.55, 1.15) | 0.69 (0.45, 1.06) | 0.88 (0.45, 1.74) | 0.228 |
| Lignoceric | Saturated | Ref | 0.90 (0.62, 1.31) | 0.79 (0.51, 1.20) | 0.88 (0.45, 1.72) | 0.339 |
| Myristic | Saturated | Ref | 0.97 (0.66, 1.44) | 1.29 (0.82, 2.03) | 1.12 (0.54, 2.33) | 0.274 |
| Palmitic | Saturated | Ref | 1.01 (0.69, 1.48) | 1.29 (0.83, 2.00) | 1.18 (0.59, 2.36) | 0.244 |
| Stearic | Saturated | Ref. | 0.79 (0.54, 1.16) | 0.84 (0.54, 1.30) | 0.96 (0.48, 1.92) | 0.729 |
| Eicosenoic | *cis* -Mono | Ref | 1.03 (0.71, 1.50) | 0.95 (0.62, 1.47) | 1.02 (0.51, 2.03) | 0.843 |
| Nervonic | *cis* -Mono | Ref | 0.96 (0.66, 1.40) | 0.99 (0.65, 1.53) | 0.82 (0.42, 1.63) | 0.752 |
| Oleic | *cis* -Mono | Ref | 1.07 (0.73, 1.56) | 1.26 (0.81, 1.95) | 1.01 (0.51, 2.02) | 0.474 |
| Palmitoleic | *cis* -Mono | Ref | 1.73 (1.17, 2.57) | 2.17 (1.37, 3.45) | 2.20 (1.04, 4.63) | 0.002 |
| alpha-Linolenic (ALA) | Omega-3 | Ref | 0.88 (0.61, 1.28) | 1.03 (0.67, 1.58) | 0.91 (0.46, 1.82) | 0.914 |
| Docosahexaenoic (DHA) | Omega-3 | Ref | 0.97 (0.67, 1.41) | 0.96 (0.63, 1.48) | 0.75 (0.37, 1.49) | 0.552 |
| Docosapentaenoic - n3 (DPA) | Omega-3 | Ref | 1.08 (0.74, 1.56) | 1.11 (0.72, 1.70) | 1.23 (0.62, 2.42) | 0.537 |
| Eicosapentaenoic (EPA) | Omega-3 | Ref | 1.42 (0.96, 2.09) | 1.30 (0.83, 2.04) | 1.45 (0.71, 2.98) | 0.287 |
| Arachidonic (AA) | Omega-6 | Ref | 0.95 (0.65, 1.37) | 0.82 (0.54, 1.26) | 0.99 (0.50, 1.96) | 0.521 |
| Dihomo-g-linolenic (DGLA) | Omega-6 | Ref | 1.20 (0.81, 1.77) | 1.53 (0.97, 2.40) | 1.31 (0.65, 2.68) | 0.098 |
| Docosapentaenoic - n6 | Omega-6 | Ref | 1.24 (0.85, 1.82) | 1.77 (1.13, 2.78) | 1.80 (0.87, 3.73) | 0.009 |
| Docosatetraenoic | Omega-6 | Ref | 1.12 (0.76, 1.63) | 1.27 (0.81, 1.97) | 1.58 (0.76, 3.25) | 0.154 |
| Eicosadienoic | Omega-6 | Ref | 1.20 (0.83, 1.74) | 1.41 (0.92, 2.16) | 1.50 (0.76, 2.98) | 0.095 |
| gamma-Linolenic (GLA) | Omega-6 | Ref. | 1.35 (0.92, 1.96) | 1.69 (1.09, 2.61) | 2.13 (1.05, 4.32) | 0.007 |
| Linoleic (LA) | Omega-6 | Ref. | 0.67 (0.46, 0.99) | 0.75 (0.48, 1.15) | 0.62 (0.31, 1.24) | 0.186 |
| Elaidic | *trans* | Ref | 0.65 (0.43, 0.99) | 0.56 (0.35, 0.89) | 0.53 (0.26, 1.07) | 0.017 |
| Palmitelaidic | *trans* | Ref | 0.77 (0.52, 1.15) | 0.77 (0.49, 1.21) | 0.92 (0.45, 1.88) | 0.499 |
| Linoelaidic | *trans* | Ref | 0.89 (0.60, 1.33) | 0.82 (0.52, 1.29) | 0.82 (0.40, 1.68) | 0.395 |

*Logistic regression comparing high vs. low (ref.) fatty acid levels adjusted for age at recruitment, BMI, diabetes, aspirin, education, population group, and smoking history. High fatty acid levels are defined as above median serum concentration in the NCI-Maryland control population

OR = Odds Ratio, CI = Confidence Interval

*Bonferroni adjusted threshold for significance is $P_{trend}$ = 0.0021 following logistic regression

**Supplementary Table 16.** Coefficient of Variation (CV) for each of the 24 fatty acids based on the analysis of 156 blind duplicates

| Fatty acid | Fatty acid class | Average CV (%) |
|---|---|---|
| Arachidic | Saturated | 12.5% |
| Behenic | Saturated | 17.4% |
| Lignoceric | Saturated | 19.2% |
| Myristic | Saturated | 4.1% |
| Palmitic | Saturated | 2.4% |
| Stearic | Saturated | 3.1% |
| Eicosenoic | *cis* -Monounsaturated | 11.9% |
| Nervonic | *cis* -Monounsaturated | 24.3% |
| Oleic | *cis* -Monounsaturated | 2.5% |
| Palmitoleic | *cis* -Monounsaturated | 3.3% |
| alpha-Linolenic (ALA) | Omega-3 | 5.2% |
| Docosahexaenoic (DHA) | Omega-3 | 2.8% |
| Docosapentaenoic - n3 (DPA) | Omega-3 | 4.6% |
| Eicosapentaenoic (EPA) | Omega-3 | 3.6% |
| Arachidonic (AA) | Omega-6 | 2.7% |
| Dihomo-g-linolenic (DGLA) | Omega-6 | 2.9% |
| Docosapentaenoic - n6 | Omega-6 | 11.8% |
| Docosatetraenoic | Omega-6 | 9.1% |
| Eicosadienoic | Omega-6 | 7.3% |
| gamma-Linolenic (GLA) | Omega-6 | 5.0% |
| Linoleic (LA) | Omega-6 | 2.5% |
| Elaidic | *trans* -Monounsaturated | 9.5% |
| Palmitelaidic | *trans* -Monounsaturated | 17.8% |
| Linoelaidic | *trans* -Polyunsaturated | 13.6% |
| **Median CV** | | **5.1%** |

**Supplementary Table 17.** List of the 82 immuno-oncological proteins detected in serum and their related biological pathway(s)

| Protein symbol | Protein name | Uniprot ID | Immune-oncological pathway(s) |
|---|---|---|---|
| ADA | Adenosine Deaminase | P00813 | Metabolism/autophagy |
| ADGRG1 | Adhesion G-protein coupled receptor G1 | Q9Y653 | Vascular & tissue remodeling |
| ANG-1 | Angiopoietin-1 | Q15389 | Vascular & tissue remodeling |
| ANGPT2 | Angiopoietin-2 | O15123 | Vascular & tissue remodeling |
| ARG1 | Arginase-1 | P05089-1 | Suppress tumor immunity |
| CAIX | Carbonic anhydrase IX | Q16790 | Metabolism/autophagy; Vascular & tissue remodeling |
| CASP-8 | Caspase-8 | Q14790 | Apoptosis/cell killing |
| CCL17 | C-C motif chemokine 17 | Q92583 | Chemotaxis; Suppress tumor immunity |
| CCL19 | C-C motif chemokine 19 | Q99731 | Chemotaxis; Suppress tumor immunity |
| CCL20 | C-C motif chemokine 20 | P78556 | Chemotaxis; Suppress tumor immunity |
| CCL23 | C-C motif chemokine 23 | P55773 | Chemotaxis; Vascular & tissue remodeling |
| CCL3 | C-C motif chemokine 3 | P10147 | Chemotaxis |
| CCL4 | C-C motif chemokine 4 | P13236 | Chemotaxis |
| CD244 | Natural killer cell receptor 2B4 | Q9BZW8 | Promote tumor immunity |
| CD27 | CD27 antigen | P26842 | Promote tumor immunity |
| CD28 | T-cell-specific surface glycoprotein CD28 | P10747 | Promote tumor immunity |
| CD4 | T-cell surface glycoprotein CD4 | P01730 | Promote tumor immunity; Suppress tumor immunity |
| CD40 | CD40L receptor | P25942 | Promote tumor immunity |
| CD40-L | CD40 ligand | P29965 | Apoptosis/cell killing; Promote tumor immunity |
| CD5 | T-cell surface glycoprotein CD5 | P06127 | Promote tumor immunity; Suppress tumor immunity |
| CD70 | CD70 antigen | P32970 | Promote tumor immunity |
| CD83 | CD83 antigen | Q01151 | Promote tumor immunity |
| CD8A | T-cell surface glycoprotein CD8 alpha chain | P01732 | Promote tumor immunity |
| CRTAM | Cytotoxic and regulatory T-cell molecule (CRTAM) | O95727 | Promote tumor immunity |
| CSF-1 | Macrophage colony-stimulating factor 1 | P09603 | Suppress tumor immunity |
| CX3CL1 | Fractalkine | P78423 | Chemotaxis; Promote tumor immunity |
| CXCL1 | C-X-C motif chemokine 1 | P09341 | Chemotaxis; Suppress tumor immunity; Vascular & tissue remodeling |
| CXCL10 | C-X-C motif chemokine 10 | P02778 | Chemotaxis; Promote tumor immunity; Vascular & tissue remodeling |
| CXCL11 | C-X-C motif chemokine 11 | O14625 | Chemotaxis; Promote tumor immunity; Suppress tumor immunity; Vascular & tissue remodeling |
| CXCL13 | C-X-C motif chemokine 13 | O43927 | Chemotaxis; Promote tumor immunity; Suppress tumor immunity |
| CXCL5 | C-X-C motif chemokine 5 | P42830 | Chemotaxis; Suppress tumor immunity; Vascular & tissue remodeling |
| CXCL9 | C-X-C motif chemokine 9 | Q07325 | Chemotaxis; Promote tumor immunity; Vascular & tissue remodeling |
| DCN | Decorin | P07585 | Vascular & tissue remodeling |
| EGF | Pro-epidermal growth factor | P01133 | Vascular & tissue remodeling |
| FASLG | Fas antigen ligand | P48023 | Apoptosis/ cell killing |
| FGF2 | Fibroblast growth factor 2 | P09038 | Vascular & tissue remodeling |
| Gal-1 | Galectin-1 | P09382 | Suppress tumor immunity; Vascular & tissue remodeling |
| Gal-9 | Galectin-9 | O00182 | Apoptosis/cell killing; Suppress tumor immunity; Vascular & tissue remodeling |
| GZMA | Granzyme A | P12544 | Apoptosis/cell killing |
| GZMB | Granzyme B | P10144 | Apoptosis/cell killing |
| GZMH | Granzyme H | P20718 | Apoptosis/cell killing |
| HGF | Hepatocyte growth factor | P14210 | Vascular & tissue remodeling |
| HO-1 | Heme oxygenase 1 | P09601 | Metabolism/autophagy |
| ICOSLG | ICOS ligand | O75144 | Promote tumor immunity |
| IL10 | Interleukin-10 | P22301 | Suppress tumor immunity |
| IL12 | Interleukin-12 | P29459,P29460 | Vascular & tissue remodeling |
| IL12RB1 | Interleukin-12 receptor subunit beta-1 | P42701 | Promote tumor immunity |
| IL18 | Interleukin-18 | Q14116 | Promote tumor immunity; Suppress tumor immunity |
| IL4 | Interleukin-4 | P05112 | Suppress tumor immunity |
| IL5 | Interleukin-5 | P05113 | Suppress tumor immunity |
| IL6 | Interleukin-6 | P05231 | Promote tumor immunity |
| IL7 | Interleukin-7 | P13232 | Promote tumor immunity |
| IL8 | Interleukin-8 | P10145 | Chemotaxis; Suppress tumor immunity; Vascular & tissue remodeling |
| KLRD1 | Natural killer cells antigen CD94 | Q13241 | Promote tumor immunity |
| LAMP3 | Lysosome-associated membrane glycoprotein 3 | Q9UQV4 | Suppress tumor immunity |
| LAP TGF-beta-1 | Latency-associated peptide transforming growth factor b | P01137 | Suppress tumor immunity |
| MCP-1 | Monocyte chemotactic protein 1 | P13500 | Chemotaxis; Vascular & tissue remodeling |
| MCP-2 | Monocyte chemotactic protein 2 | P80075 | ChemotaxisChemotaxisChemotaxis |
| MCP-3 | Monocyte chemotactic protein 3 | P80098 | Chemotaxis |
| MCP-4 | Monocyte chemotactic protein 4 | Q99616 | Chemotaxis |
| MIC-A/B | MHC class I polypeptide-related sequence A/B | Q29983,Q29980 | Suppress tumor immunity |
| MMP12 | Matrix metalloproteinase-12 | P39900 | Suppress tumor immunity; Vascular & tissue remodeling |
| MMP7 | Matrix metalloproteinase-7 | P09237 | Apoptosis/cell killing; Suppress tumor immunity |
| NCR1 | Natural cytotoxicity triggering receptor 1 | O76036 | Promote tumor immunity |
| NOS3 | Nitric oxide synthase, endothelial | P29474 | Vascular & tissue remodeling |
| PD-L1 | Programmed cell death 1 ligand 1 | Q9NZQ7 | Suppress tumor immunity |
| PD-L2 | Programmed cell death 1 ligand 2 | Q9BQ51 | Suppress tumor immunity |
| PDCD1 | Programmed cell death protein 1 | Q15116 | Suppress tumor immunity |
| PDGF subunit B | Platelet-derived growth factor subunit B | P01127 | Vascular & tissue remodeling |
| PGF | Placenta growth factor | P49763 | Vascular & tissue remodeling |
| PTN | Pleiotrophin | P21246 | Vascular & tissue remodeling |
| TIE2 | Angiopoietin-1 receptor | Q02763 | Vascular & tissue remodeling |
| TNFRSF12A | Tumor necrosis factor receptor superfamily member 12A | Q9NP84 | Apoptosis/cell killing; Vascular & tissue remodeling |
| TNFRSF21 | Tumor necrosis factor receptor superfamily member 21 | O75509 | Apoptosis/cell killing |
| TNFRSF4 | Tumor necrosis factor receptor superfamily member 4 | P43489 | Promote tumor immunity |
| TNFRSF9 | Tumor necrosis factor receptor superfamily member 9 | Q07011 | Promote tumor immunity |
| TNFSF14 | Tumor necrosis factor ligand superfamily member 14 | O43557 | Promote tumor immunity |
| TRAIL | TNF-related apoptosis-inducing ligand | P50591 | Apoptosis/cell killing |
| TWEAK | Tumor necrosis factor (Ligand) superfamily, member 12 | O43508 | Apoptosis/cell killing; Vascular & tissue remodeling |
| VEGFA | Vascular endothelial growth factor A | P15692 | Vascular & tissue remodeling |
| VEGFC | Vascular endothelial growth factor C | P49767 | Vascular & tissue remodeling |
| VEGFR-2 | Vascular endothelial growth factor receptor 2 | P35968 | Vascular & tissue remodeling |
