# Supplementary material for: Circulating trans fatty acids are associated with prostate cancer in Ghanaian and American men
Source: Nat Commun. 2023 Jul 19;14:4322. doi: 10.1038/s41467-023-39865-9 (PMC10356769; doi:10.1038/s41467-023-39865-9)
Supplement: Supplementary file 3 — Description of Additional Supplementary Files [file 41467_2023_39865_MOESM3_ESM.pdf]

Description of Additional Supplementary Files

Supplementary Data 1. Full regression results from multivariable linear regression tests that evaluate the association of 24 fatty acids with age, BMI, education, smoking, diabetes, and aspirin use in Ghanaian, African American, and European American controls and cases.

Supplementary Data 2. Full regression results from multivariable linear regression tests that evaluate the association of 24 fatty acids with age, BMI, education, smoking, diabetes, aspirin use, and Gleason score in Ghanaian, African American, and European American cases.

Supplementary Data 3. Survey data on dietary intake of participants in the NCI Maryland Prostate Cancer Case-Control Study.

Supplementary Data 4. The fraction of variance in each of the fatty acids explained by dietary factors in African American and European American population controls.

Supplementary Data 5. The fraction of variance in each of the fatty acids explained by dietary factors in African American and European American cases.
