# Supplementary material for: Circulating trans fatty acids are associated with prostate cancer in Ghanaian and American men
Source: Nat Commun. 2023 Jul 19;14:4322. doi: 10.1038/s41467-023-39865-9 (PMC10356769; doi:10.1038/s41467-023-39865-9)
Supplement: Supplementary file 9 — Reporting Summary [file 41467_2023_39865_MOESM9_ESM.pdf]

nature portfolio

Corresponding author(s): Stefan Ambs

Last updated by author(s): Jun 29, 2023

Reporting Summary

Nature Portfolio wishes to improve the reproducibility of the work that we publish. This form provides structure for consistency and transparency in reporting. For further information on Nature Portfolio policies, see our Editorial Policies and the Editorial Policy Checklist.

Statistics

For all statistical analyses, confirm that the following items are present in the figure legend, table legend, main text, or Methods section.

n/a	Confirmed	
☐	☒	The exact sample size (*n*) for each experimental group/condition, given as a discrete number and unit of measurement
☐	☒	A statement on whether measurements were taken from distinct samples or whether the same sample was measured repeatedly
☐	☒	The statistical test(s) used AND whether they are one- or two-sided<br>*Only common tests should be described solely by name; describe more complex techniques in the Methods section.*
☐	☒	A description of all covariates tested
☐	☒	A description of any assumptions or corrections, such as tests of normality and adjustment for multiple comparisons
☐	☒	A full description of the statistical parameters including central tendency (e.g. means) or other basic estimates (e.g. regression coefficient) AND variation (e.g. standard deviation) or associated estimates of uncertainty (e.g. confidence intervals)
☐	☒	For null hypothesis testing, the test statistic (e.g. *F*, *t*, *r*) with confidence intervals, effect sizes, degrees of freedom and *P* value noted<br>*Give P values as exact values whenever suitable.*
☒	☐	For Bayesian analysis, information on the choice of priors and Markov chain Monte Carlo settings
☐	☒	For hierarchical and complex designs, identification of the appropriate level for tests and full reporting of outcomes
☐	☒	Estimates of effect sizes (e.g. Cohen's *d*, Pearson's *r*), indicating how they were calculated

Our web collection on statistics for biologists contains articles on many of the points above.

Software and code

Policy information about availability of computer code

Data collection

We did not use a specific software to collect data for the study.

Data analysis

Code availability: The scripts used in our bioinformatics pipeline to perform data analysis and visualization are available as a public GitHub repository at https://github.com/tsionzminas/Prostate-Cancer-and-Circulating-Fatty-acids or or at Zenodo under the accession code 8023186 [https://doi.org/10.5281/zenodo.8023186]

- Data analyses were performed using Stata/SE 16.0, JMP 14.0, and R statistical packages.
- Single nucleotide polymorphism genotype data were generated using the Infinium HumanOmniS-Quad BeadChip array and analyzed using the "High-Throughput Workflow" section on the standard Illumina microarray data analysis workflow page. Genotype calls initially output as Genotype Call files (.gtc) are converted to PLINK 1.9 using the Illumina-provided open-source library (github.com/Illumina/BeadArrayFiles).
- Heat maps and dendrograms showing unsupervised hierarchical clustering of 24 individual fatty acids were generated using JMP 14.0.
- The association of age, body mass index (BMI), education, aspirin use, smoking, and diabetes with absolute concentrations of circulating fatty acids (as continuous value) was assessed by means of multivariable linear regression models implemented by the function lm in the base R package stats (version 3.6.1).
- Variance analysis for the levels of each of the 24 fatty acids were simultaneously assessed as a function of demographic, clinical, and genetic factors in men with prostate cancer from the NCI-Maryland and NCI-Ghana studies. The analysis was implemented by the function aov in the base R package stats (version 3.6.1).
- Unconditional logistic regression was used to compute the odds ratios (OR) and 95% confidence intervals (CI) to assess the association of circulating levels of fatty acids with prostate cancer.
- Student's t-tests were used to compare fatty acid mean concentrations by population group.

- An association was considered statistically significant with P< 0.05 or Bonferroni-corrected significance threshold in instances where correction for multiple testing was required.

For manuscripts utilizing custom algorithms or software that are central to the research but not yet described in published literature, software must be made available to editors and reviewers. We strongly encourage code deposition in a community repository (e.g. GitHub). See the Nature Portfolio guidelines for submitting code & software for further information.

Data

Policy information about availability of data

All manuscripts must include a data availability statement. This statement should provide the following information, where applicable:

- Accession codes, unique identifiers, or web links for publicly available datasets
- A description of any restrictions on data availability
- For clinical datasets or third party data, please ensure that the statement adheres to our policy

Clinical, demographic and molecular data used for this study (i.e., self-reported race, age, BMI, education, aspirin use, diabetes, smoking status, NCCN risk score, proteomics data, GWAS data, and fatty acid data) are deposited in the Open Science Framework database (https://osf.io/tscgh/) under the accession code TSCGH [https://doi.org/10.17605/OSF.IO/TSCGH] and as a public GitHub repository at https://github.com/tsionzminas/Prostate-Cancer-and-Circulating-Fatty-acids or at Zenodo under the accession code 8023186 [https://doi.org/10.5281/zenodo.8023186]. The full proteomics data was deposited in the Open Science Framework database under the accession code 327HA [https://doi.org/10.17605/OSF.IO/327HA]. Source data are provided with this paper. The remaining data are available within the paper, Supplementary Information, and Supplementary Data.

Research involving human participants, their data, or biological material

Policy information about studies with human participants or human data. See also policy information about sex, gender (identity/presentation), and sexual orientation and race, ethnicity and racism.

Reporting on sex and gender

Participants in the study self-reported as "male" gender in the study questionnaires when given the choice between male and female gender. Given the focus of this study is on prostate cancer, which only applies to the male sex, the study design was restricted to only individuals who indicated "male" on their study questionnaire and are referred to as "men" in the manuscript. Disaggregated sex and gender data were not collected for this study. Patient informed consent has been provided for sharing de-identified individual-level data. 2,934 men were included in this study. Sex- and gender-based analyses were not performed due to the study being restricted to only one sex.

Reporting on race, ethnicity, or other socially relevant groupings

Participants were categorized into population groups based on self-report in the study questionnaires. Racial categorizations included White/Caucasian, Black/African American, Asian, Native Hawaiian/Other Pacific Islander, American Indian/Alaska Native for the NCI-Maryland Prostate Cancer Case Control Study and African, African American, and European American for the NCI-Ghana Prostate Cancer Case Control Study. The terms Ghanaian, African American, and European American are used as population group categorizations throughout the manuscript to capture genetic variation related to fatty acid and immune profiles. The terms "White" and "Black" were not used due to their relationship with socioeconomic-related racial groupings, which were not formally explored in this manuscript. Population specific differences in fatty acid concentrations was one of the main focuses of this manuscript, so analyses were either stratified by population group or population group was controlled for in statistical models when analyzing total population data.

Population characteristics

Population characteristics

Participants in the NCI-Maryland Prostate Cancer Case-Control study included 1,691 African American (AA) and European American (EA) men of whom 846 were cases and 845 were controls. The NCI-Ghana Prostate Cancer Case-Control study included 1,243 men of whom 585 were cases and 658 were controls. In our analyses, we investigated the association of age at study entry, body mass index (BMI), education, aspirin use, smoking, diabetes, and prostate cancer with the levels of the 24 circulating fatty acids.

In the NCI-Maryland study, cases were slightly younger than controls. The median age at study entry of cases was 64 years (AA: 63 years vs. EA: 65 years). The median age at study entry of controls was 65 years (AA:64 years vs. EA: 66.5 years). Cases and controls had similar BMI distributions. The mean BMI of cases was 28.0kg/mA2 (AA: 28kg/mA2 vs. EA: 28kg/mA2) whereas the mean BMI of controls was 28.7kg/mA2 (AA: 29.7kg/mA2 vs. EA: 27.8kg/mA2). In addition, 24% of cases were current smokers (AA: 33% vs. EA: 15%) compared to 13% of controls (AA: 18% vs. EA: 9%). More cases than controls attained only high school or less {36% vs. 23.1%). 58% of controls (AA: 52% vs. EA: 62%) vs. 49% of cases (AA: 42% vs. EA: 56%) were regular aspirin users. 21% of cases (AA: 28% vs. EA. 15%) compared to 23% of controls (AA: 30% vs. EA: 17%) were diabetic. 20 % of cases (AA: 8% vs. EA: 30%) whereas 32% of controls (AA: 23% vs. EA: 38%) had annual household income greater than $90,000. High Gleason score (>7) was reported in 146 out of the 846 patients (17%) (AA: 17% vs. EA: 17%). 47 out of the 846 patients {6%) had regional or distant metastasis (AA: 6% vs. EA: 5%).

In the NCI-Ghana study, cases were older than controls with median age at study entry of 70 years vs. 59 years. The mean BMI for cases was 25.6 kg/mA2 whereas the mean BMI for controls was 24.3 kg/mA2. In addition, 2% of cases were current smokers compared to 14% of controls. 58% of cases have attended only high school or less compared to 83% of controls. Moreover, 16% of cases vs. 10% of controls were regular aspirin users. More cases than controls were diabetic {18% vs.7%). High Gleason score(> 7) was reported in 197 out of the 585 cases {34%).

Recruitment

Prior to study enrollment, all participants signed an informed consent.

For the NCI-Maryland study, cases were recruited at the Baltimore Veterans Affairs Medical Center and the University of Maryland Medical Center. A total of 976 cases (489 AA and 487 EA men) were recruited into this study between 2005 and

2015. Controls were identified through the Maryland Department of Motor Vehicle Administration database and were frequency-matched to cases on age and race. A total of 1,033 population controls were recruited (485 AA and 548 EA men). At the time of enrollment, both cases and controls were administered a survey by a trained interviewer and a blood sample was collected. Participants were compensated up to 50 USD for their involvement and participation in this study.

For the NCI-Ghana study, prostate cancer cases were recruited at Korie Bu Teaching Hospital in Accra, Ghana between 2008 and 2012. The cases were diagnosed using Digital Rectal Exam (DRE) and PSA tests, followed by biopsy confirmation. Immediately after diagnosis and before treatment, cases were consented and asked to submit blood specimen and questionnaire data. Controls were identified through probability sampling using the 2000 Ghana Population and Housing Census data to recruit approximately 1,000 men aged 50-74 years in the Greater Accra region between 2004 and 2006. These men were confirmed to not have prostate cancer by PSA testing and DRE. Participants were compensated up to 5 USD for transportation costs related to the study.

Case-Control studies are susceptible to selection and recall biases. A selection bias in the case vs. control analysis can occur when cases and controls are not recruited from the same target population. This bias typically arises when selection criteria are associated with the risk factor under investigation. A recall bias would occur when cases and controls would recall differently the exposure to a risk factor which usually occurs when the participants are aware that the exposure to a factor may cause the disease. We do not think that these biases affected our findings because in our study we did not perform case versus control comparisons, nor would the participants assume associations of the self-reported exposures with the measured immune-oncology markers. All analyses were either case-only or control-only analyses. Comparisons focused on differences between European American, African American, and Ghanaian men. Yet the men recruited in the United States and Ghana may represent different population groups, and we cannot exclude that the collection of blood samples in Ghana may have affected the measurement of the immune-oncology differently than the collection of the samples in the United States although similar protocols were used.

In both studies, patients were approached at the participating hospitals after a prostate cancer diagnosis and then recruited. Cases at all stages of disease were recruited. We are not aware of any biases in patient recruitment but cannot exclude a referral bias.

Both studies recruited population-based controls. For the NCI-Maryland study, these controls had double eligibility for the prostate cancer study and a lung cancer study. Controls were age 40-90 and without a history of cancer other than non-melanomic skin cancer, had a residential working phone number, were born in the United States, and spoke English well enough to be interviewed. Controls were not eligible when they had a history of radiotherapy or chemotherapy, or when they were severely ill or resided in an institution. A previous analysis showed a 91% participation rate among those who could be reached by phone and were eligible for the study. These controls were similar to the general Maryland population in terms of education levels, smoking status, and body mass index. For the NCI-Ghana study, controls were age 50-74 and were randomly selected using Ghana Census Bureau data. Men too sick to participate were excluded, and also those men with screening detected prostate cancer (biopsy and PSA-based). The participation rate was 98.8% prior to screening for prostate cancer.

Ethics oversight

The NCI-Maryland Prostate Cancer Case Control Study protocol was approved by the National Cancer Institute (protocol # 05-C-N021) and the University of Maryland (protocol #0298229) Institutional Review Boards. The NCI-Ghana Prostate Cancer Case Control Study protocol was approved by Institutional Review Boards at the University of Ghana (protocol #001/01-02) and at the National Cancer Institute (protocol #02CN240).

Note that full information on the approval of the study protocol must also be provided in the manuscript.

Field-specific reporting

Please select the one below that is the best fit for your research. If you are not sure, read the appropriate sections before making your selection.

☒ Life sciences ☐ Behavioural & social sciences ☐ Ecological, evolutionary & environmental sciences

For a reference copy of the document with all sections, see nature.com/documents/nr-reporting-summary-flat.pdf

Life sciences study design

All studies must disclose on these points even when the disclosure is negative.

Sample size

No sample size calculation was performed since this study was based on already completed case-control studies. We have included all individuals whose serum samples were available into the study herein.

Data exclusions

None

Replication

To verify our experimental findings, we have utilized two case-control studies with large representations of men of African ancestry: the NCI-Ghana and NCI-Maryland Prostate Cancer Case-Control Studies. All analyses were successfully replicated independently by two data scientists using the provided datasets.

Randomization

We assayed 24 fatty acids in a total of 3,092 serum samples including 156 (5%) randomly selected blinded duplicates. To control for any batch effects, the serum samples were assayed in a random order along with the 5% blind duplicates. Hundred percent of the samples passed a stringent quality control.

Blinding

Omegaquant, the company that assayed the 24 fatty acids in 3092 samples, was blinded to the 5% duplicates that we have randomized along

Blinding	the 3092 samples. The median Coefficient of Variation (CV) calculated based on the 156 blind duplicates was 5.1% across the 24 fatty acids

Reporting for specific materials, systems and methods

We require information from authors about some types of materials, experimental systems and methods used in many studies. Here, indicate whether each material, system or method listed is relevant to your study. If you are not sure if a list item applies to your research, read the appropriate section before selecting a response.

Materials & experimental systems

n/a	Involved in the study
☒	☐ Antibodies
☒	☐ Eukaryotic cell lines
☒	☐ Palaeontology and archaeology
☒	☐ Animals and other organisms
☒	☐ Clinical data
☒	☐ Dual use research of concern
☒	☐ Plants

Methods

n/a	Involved in the study
☒	☐ ChIP-seq
☒	☐ Flow cytometry
☒	☐ MRI-based neuroimaging
